# Cromolyn sodium delays disease onset and is neuroprotective in the SOD1^*G93A*^ Mouse Model of amyotrophic lateral sclerosis

**DOI:** 10.1038/s41598-019-53982-w

**Published:** 2019-11-27

**Authors:** Eric J. Granucci, Ana Griciuc, Kaly A. Mueller, Alexandra N. Mills, Hoang Le, Amanda M. Dios, Danielle McGinty, Joao Pereira, David Elmaleh, James D. Berry, Sabrina Paganoni, Merit E. Cudkowicz, Rudolph E. Tanzi, Ghazaleh Sadri-Vakili

**Affiliations:** 1 Healey Center for ALS at MassGeneral Hospital, Massachusetts General Hospital, Boston, MA USA; 2 Genetics and Aging Research Unit, McCance Center for Brain Health, MassGeneral Institute for Neurodegenerative disease, Department of Neurology, Massachusetts General Hospital and Harvard Medical School, Charlestown, MA 02129 USA; 3 AZTherapies, Boston, MA USA; 4 Spaulding Rehabilitation Hospital, Department of PM&R, Harvard Medical School, Boston, MA USA

**Keywords:** Neuromuscular disease, Amyotrophic lateral sclerosis

## Abstract

Accumulating evidence suggests that neuroinflammatory processes are implicated in the initiation and progression of amyotrophic lateral sclerosis (ALS). Previous reports have demonstrated an increase in microgliosis and astrogliosis in the lumbar spinal cord of SOD1^*G93A*^ transgenic mice before the onset of symptoms, a neuroinflammatory response which correlated with disease progression. Importantly, early stage homeostatic microglia enhanced motor neuron survival, while pro-inflammatory microglia were toxic to motor neurons in the SOD1^*G93A*^ mice. Recent studies from our group have demonstrated that cromolyn sodium, an FDA approved compound, exerts neuroprotective effects in mouse models of Alzheimer’s disease by altering microglial cell activation. Here, we tested the neuroprotective and anti-inflammatory effects of cromolyn sodium in the SOD1^*G93A*^ mouse model of ALS. Our results indicate that cromolyn sodium treatment significantly delayed the onset of neurological symptoms, and improved deficits in PaGE performance in both male and female mice, however, there was only an effect on survival in female mice. Furthermore, there was a significant increase in motor neuron survival in the lumbar spinal cord as well as a significant decrease in the denervation of the neuromuscular junction of the tibialis anterior muscle in cromolyn treated transgenic SOD1^*G93A*^ mice. Lastly, cromolyn treatment decreased the expression of pro-inflammatory cytokines/chemokines in the lumbar spinal cord and plasma and decreased mast cell degranulation in the tibialis anterior muscle of transgenic SOD1^*G93A*^ mice. Together, these findings suggest that cromolyn sodium provides neuroprotection in the SOD1^*G93A*^ mice by decreasing the inflammatory response.

## Introduction

Although the etiology of amyotrophic lateral sclerosis (ALS), a disabling and fatal neurodegenerative disease, is not yet fully understood, accumulating evidence suggests that neuroinflammatory processes are implicated in the initiation and progression of disease^1,2^. This neuroinflammation is mediated, at least in part, by resident central nervous system (CNS) immune cells, i.e. activated microglia and reactive astrocytes, which produce reactive oxygen species, nitric oxide, and pro-inflammatory cytokines leading to neuronal cell death^3–5^. Microglial cells can assume activation states in a spectrum from the anti-inflammatory to the pro-inflammatory. Activated microglia were detected near motor neurons of transgenic SOD1^*G93A*^ (TgSOD1^*G93A*^) mice at 39 days, well before the onset of weakness, and this neuroinflammatory response correlated with disease progression^6^. In addition, microglia from ALS mice produced more harmful oxidative species than wild-type microglia, suggesting a deleterious role for microglia in ALS^7^. Moreover, early stage anti-inflammatory microglia enhanced motor neuron survival, while pro-inflammatory microglia were toxic to motor neurons in the TgSOD1^*G93A*^ mice^8^. Together, these findings suggest that during ALS progression, there is a shift from the anti-inflammatory and neuroprotective to the pro-inflammatory and neurotoxic microglial activation state^8^.

Given that microglia- and astrocyte-induced neuroinflammation is linked to excessive neuronal cell death in ALS, one therapeutic approach would be to use pharmacological agents that convert microglial cells from the pro-inflammatory to an anti-inflammatory and neuroprotective state. Recent studies from our group have demonstrated that cromolyn sodium, an FDA-approved compound used for the treatment of asthma, exerts neuroprotective effects in cellular and animal models of Alzheimer’s disease (AD)^9,10^. Specifically, cromolyn treatment significantly inhibited amyloid beta (Aβ) aggregation *in vitro* and reduced the concentration of soluble monomeric Aβ in the transgenic APPswe/PS1σE9 mouse model of AD^9^. Furthermore, *in vivo* microdialysis studies demonstrated that the half-life of Aβ was significantly reduced in cromolyn treated mice^9^. Our more recent findings probed the mechanisms whereby Aβ accumulation was decreased in response to cromolyn and demonstrated that cromolyn alone or in combination with ibuprofen, led to decreased levels of insoluble Aβ40 and Aβ42 in the Tg2576 mouse model of AD^10^. Importantly, the percentage of Iba1^+^ microglia that co-localized with Aβ plaques was significantly increased following cromolyn treatment, suggesting that cromolyn promoted microglial clustering around Aβ plaques and resulted in the subsequent uptake and removal of Aβ^10^. Lastly, microglial cell cultures treated with cromolyn exhibited increased Aβ uptake compared to vehicle treated cells^10^. Collectively, these results demonstrate that cromolyn treatment reduced aggregation-prone Aβ levels and induced an anti-inflammatory microglial activation state that leads to Aβ uptake and clearance.

Given these promising findings in AD, we investigated the neuroprotective efficacy of cromolyn sodium treatment in the SOD1^*G93A*^ mouse model of ALS. Male and female Wild-type (Wt) and Tg SOD1^*G93A*^ mice were treated with cromolyn via intraperitoneal injection starting post-natal day 60 (P60) until euthanasia. Alterations in behavior and neuropathological markers such as body weight, neurological score, motor deficits, survival, and lumbar spinal cord motor neuron counts were assessed following treatment. Additionally, we assessed the effects of cromolyn on neuromuscular junction (NMJ) integrity and innervation of the tibialis anterior muscle. Lastly, we investigated the effects of cromolyn treatment on inflammation by assessing astrogliosis and microgliosis in the lumbar spinal cord, levels of pro-inflammatory cytokines and chemokines in the spinal cord and plasma, and mast cell numbers and degranulation in the tibialis anterior muscle.

## Results

A total of 149 male and female age- and litter-matched transgenic (Tg) SOD1^*G93A*^ and wild-type (Wt) mice were used with the following breakdown: Females (19 Wt-Vehicle, 17 Wt-Cromolyn, 19 TgSOD1-Vehicle, and 17 TgSOD1-Cromolyn) and Males (18 Wt-Vehicle, 21 Wt-Cromolyn, 21 TgSOD1-Vehicle, 17 TgSOD1-Cromolyn). The mice received once daily injections of either vehicle or cromolyn sodium (6.3 mg/kg, i.p.) 5 days per week starting at P60 until euthanasia. This treatment regimen was chosen based on our previous studies in the AD mice^9,10^ together with the knowledge that early immunoregulatory treatment is necessary to successfully interrupt ALS-induced neuroinflammation^8,11^.

### Cromolyn sodium treatment delayed disease onset in TgSOD1 mice

We assessed the effects of cromolyn treatment on disease onset by first measuring alterations in neurological score. We used the criteria from ALS TDI^12,13^ which define neurological score as follows:

Score of 0: Full extension of hind legs away from lateral midline when mouse is suspended by its tail, and mouse can hold this for two seconds, suspended two to three times.

Score of 1: Collapse or partial collapse of leg extension towards lateral midline (weakness) or trembling of hind legs during tail suspension.

Score of 2: Toes curl under at least twice during walking of 12 inches, or any part of foot is dragging along cage bottom/table.

Score of 3: Rigid paralysis or minimal joint movement, foot not being used for generating forward motion.

Score of 4: Mouse cannot right itself within 10 seconds after being placed on either side.

**Figure 1:**
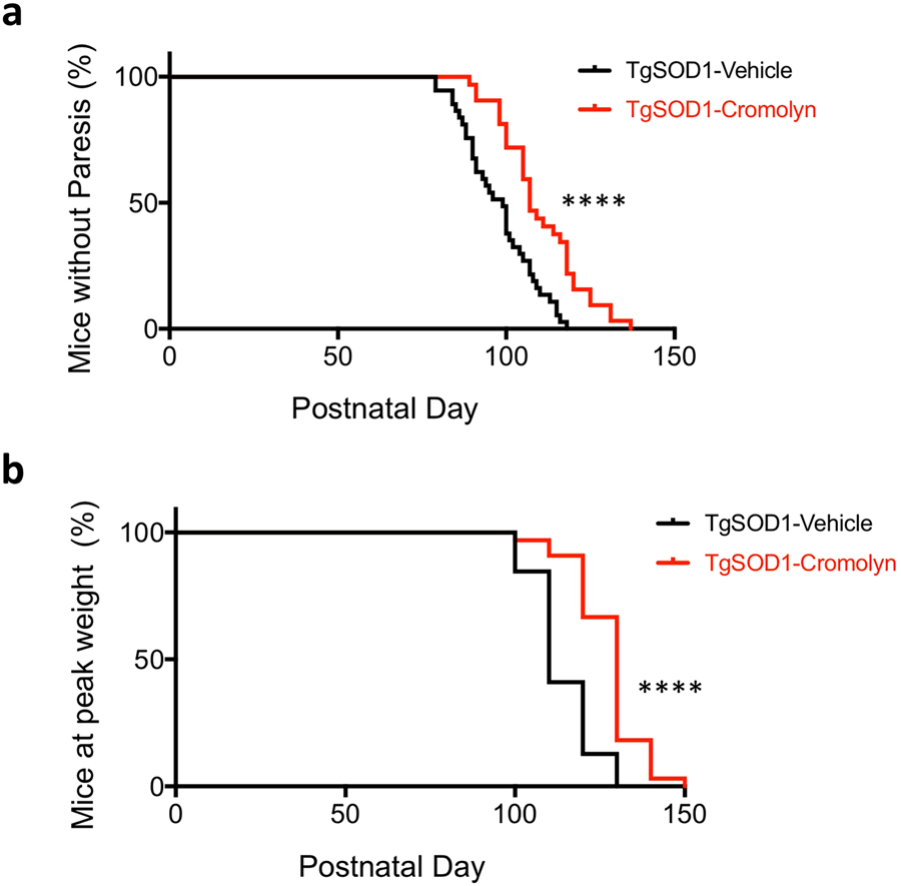
Cromolyn sodium treatment delayed disease onset in TgSOD1 mice. (**a**) There was a significant effect of cromolyn treatment on the onset of motor symptoms as measured by age at paresis onset (Mantel-Cox test), with a median age of onset of 99 days for TgSOD1-Vehicle group and 107 days for the TgSOD1-Cromolyn group. (**b**) There was a significant difference in peak body weight between TgSOD1-Vehicle compared to TgSOD1-Cromolyn group as measured by Mantel-Cox test, with median age of onset of 110 days for TgSOD1-Vehicle group and 130 days for the TgSOD1-Cromolyn group. TgSOD1-Vehicle (n = 40; black), and TgSOD1-Cromolyn (n = 34; red). *Denotes differences between TgSOD1-Vehicle and Tg-SOD1-Cromolyn. ****p < 0.0001.

There was a significant effect of cromolyn treatment on the onset of motor symptoms as measured by age at paresis onset, when the mice reached a neurological score of 2 (Mantel-Cox test, p < 0.0001), with a median age of onset of 99 days for TgSOD1-Vehicle group and 107 days for the TgSOD1-Cromolyn group (Fig. 1a). In addition, we assessed the effects of cromolyn on disease onset by determining the effects of cromolyn on peak body weight. There was a significant effect of treatment on peak body weight (Mantel-Cox test, p < 0.0001), with a median age of onset of 110 days for TgSOD1-Vehicle group and 130 days for the TgSOD1-Cromolyn group (Fig. 1b). These findings suggest that cromolyn treatment delayed disease onset in TgSOD1 mice. Male and female data are shown separately in Fig, S1a,b. Both female mice (Mantel-Cox test, p = 0.0009) (Fig. S1a) and male mice (Mantel-Cox test, p = 0.0193) (Fig. S1b) demonstrated a significant delay in the onset of motor symptoms following cromolyn treatment.

We also assessed the effect of cromolyn sodium treatment on body weight in each group. Two-way ANOVA demonstrated a significant effect of age [F(9, 1143) = 10.58, p < 0.0001], treatment [F(3, 1143) = 47.99, p < 0.0001], and age X treatment interaction effect [F(27, 1143) = 4.578, p < 0.0001] on body weight. Tukey’s multiple comparison test revealed that there was a significant decrease in body weight in the TgSOD1-Vehicle group compared to both Wt-Vehicle and Wt-Cromolyn at P100, P110, P120, P130, P140, and P150 (Fig. S2a). There was also a significant decrease in body weight in the TgSOD1-Cromolyn group compared to Wt-Cromolyn group at P100, P110, P120, P130, P140, and P150. There was a significant difference in body weight between the TgSOD1-Cromolyn group and Wt-Vehicle group at P120, P130, and P140 only. Importantly, there was a significant improvement in body weight in the TgSOD1-Cromolyn group compared to TgSOD1-Vehicle group at P130, suggesting that cromolyn treatment delayed body weight loss in the treated mice at this timepoint (Fig. S2a). Male and female data are shown separately in Fig. S2b,c and statistical analysis is provided in Supplementary Data.

### Cromolyn sodium treatment improved performance on PaGE task but did not alter rotarod or gait performance

**Figure 2:**
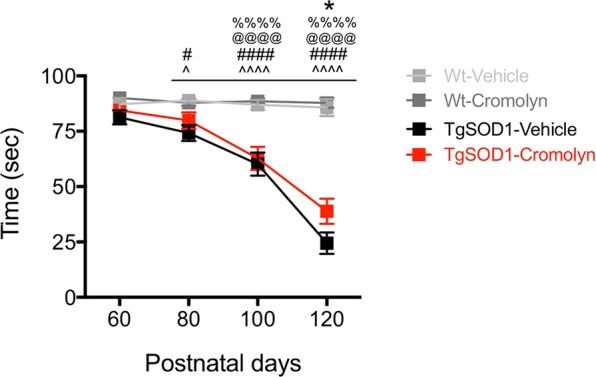
Cromolyn sodium treatment improved performance on the PaGE task in TgSOD1 mice. Two-way ANOVA and Tukey’s post-hoc analysis revealed a significant improvement in PaGE performance in TgSOD1-Cromolyn compared to TgSOD1-Vehicle group at P120 and P140. There was a significant decrease in PaGE in the TgSOD1-Vehicle group at P80, P100, P120, and P140 compared to Wt-Vehicle and Wt-Cromolyn groups. In addition, there was a significant decrease in PaGE in the TgSOD1-Cromolyn group compared to both wild-type groups at P100 and P120. Wt-Vehicle (n = 37; light grey), Wt-Cromolyn (n = 38; dark grey), TgSOD1-Vehicle (n = 40; black), and TgSOD1-Cromolyn (n = 34; red). Data are presented as median and interquartile ranges. *Denotes differences between TgSOD1-Vehicle and Tg-SOD1-Cromolyn; ^^^denotes differences between TgSOD1-Vehicle and Wt-Vehicle; ^#^denotes differences between TgSOD1-Vehicle and Wt-Cromolyn; ^@^denotes differences between TgSOD1-Cromolyn and Wt-Vehicle; ^%^denotes differences between TgSOD1-Cromolyn and Wt-Cromolyn. *p < 0.05; ****p < 0.0001, the same statistical significance is associated with each symbol. Data are presented as median and interquartile ranges.

The effect of cromolyn sodium treatment was also assessed on alterations in muscle strength using the paw grip endurance (PaGE) task. Two-way ANOVA demonstrated a significant effect of age [F(3, 476) = 41.40, p < 0.0001], treatment [F(3, 476) = 68.18, p < 0.0001] and a significant age X treatment interaction on PaGE [F(9, 476) = 12.72, p < 0.0001] (Fig. 2). Tukey’s post-hoc analysis revealed a significant decrease in PaGE in the TgSOD1-Vehicle group at P80, P100, and P120 compared to Wt-Vehicle and Wt-Cromolyn groups (Fig. 2). In addition, there was a significant decrease in PaGE performance in the TgSOD1-Cromolyn group compared to both Wt groups at P100 and P120. Importantly, there was a significant improvement in PaGE performance in TgSOD1-Cromolyn compared to TgSOD1-Vehicle group at P120 (Fig. 2). Thus, cromolyn treatment improved PaGE performance in treated TgSOD1 mice as compared to the vehicle treated TgSOD1 group. Male and female data are shown separately in Fig. S3a,b.

Motor coordination was assessed using the rotarod test. Two-way ANOVA demonstrated a significant effect of age [F(2, 361) = 34.49, p < 0.0001] and treatment [F(3, 361) = 42.25, p < 0.0001]. However, there was no significant age X treatment interaction on rotarod performance [F(6, 361) = 0.704, p = 0.646]. Tukey’s post-hoc analysis revealed a significant difference between TgSOD1-Vehicle and both Wt-Vehicle as well as Wt-Cromolyn at P70, P90 and P120 (Fig. S4a). Similarly, post-hoc analysis revealed a significant decrease in rotarod performance between TgSOD1-Cromolyn group compared to Wt-Vehicle and Wt-Cromolyn at all time points (Fig. S4a). However, there was no difference in rotarod performance between the TgSOD1-Vehicle and TgSOD1-Cromolyn mice. These data indicate that cromolyn treatment did not alter rotarod performance in TgSOD1 mice. Male and female data are shown separately in Fig. S4b,c, please see Supplementary Data for statistical analysis.

The effect of cromolyn treatment was also assessed on gait performance by measuring stride length and width. Two-way ANOVA demonstrated a significant effect of age [F(2, 403) = 62.78, p < 0.0001], treatment [F(3, 403) = 18.96, p < 0.0001], and age X treatment interaction on stride length [F(6, 403) = 16.99, p < 0.0001] in all groups. Tukey’s post-hoc analysis revealed a significant decrease in stride length in TgSOD1-Vehicle compared with both Wt groups at P120 (Fig. S5a). Similarly, post-hoc analysis revealed a significant decrease in stride length in TgSOD1-Cromolyn group compared to wild-type mice at P120 (Fig. S5a) suggesting that cromolyn treatment had no effect on stride length. In addition, alterations in stride width were also assessed following treatment. In all groups, two-way ANOVA revealed that while there was no effect of treatment, there was a significant effect of age [F(2, 397) = 18.3, p < 0.0001] and age X treatment [F(6, 397) = 3.159, p = 0.0049] on stride width. Tukey’s post-hoc analysis revealed a significant increase in stride width at P120 in TgSOD1-Vehicle group compared to Wt-Vehicle (Fig. S6a). Thus, cromolyn treatment did not alter gait performance (e.g. stride length or width) in TgSOD1 mice. Male and female data are shown separately in Figs S5b,c and S6b,c, please see Supplementary Data for statistical analysis.

### Effect of cromolyn sodium treatment on survival

**Figure 3:**
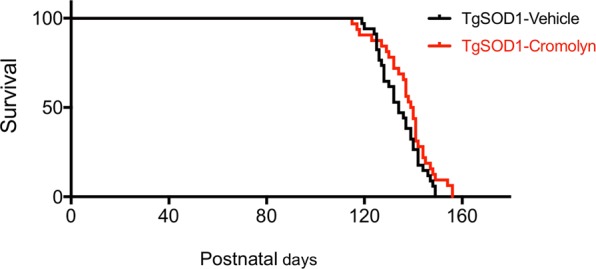
Cromolyn sodium does not alter survival in TgSOD1 mice. Cromolyn treatment did not have a significant effect on survival in cromolyn treated mice (Mantel-Cox). TgSOD1-Vehicle (n = 40; black), and TgSOD1-Cromolyn (n = 34; red). Data are presented as median and interquartile ranges.

We also assessed the effect of treatment on survival and found that there was no significant effect on survival in treated mice (Mantel-Cox test, p = 0.1096) (Fig. 3). While cromolyn treatment did not have a significant effect on survival in all treated mice (Mantel-Cox test, p = 0.1096) or male mice alone (Mantel-Cox test, p < 0.8831) (Figs 3 and S7b), there was a significant effect of treatment on female survival (Mantel-Cox test, p = 0.01) (Fig. S7a). These results suggest that cromolyn treatment only increases survival in female TgSOD1 mice.

### Cromolyn treatment is neuroprotective and increases survival of motor neurons in the lumbar spinal cord

**Figure 4:**
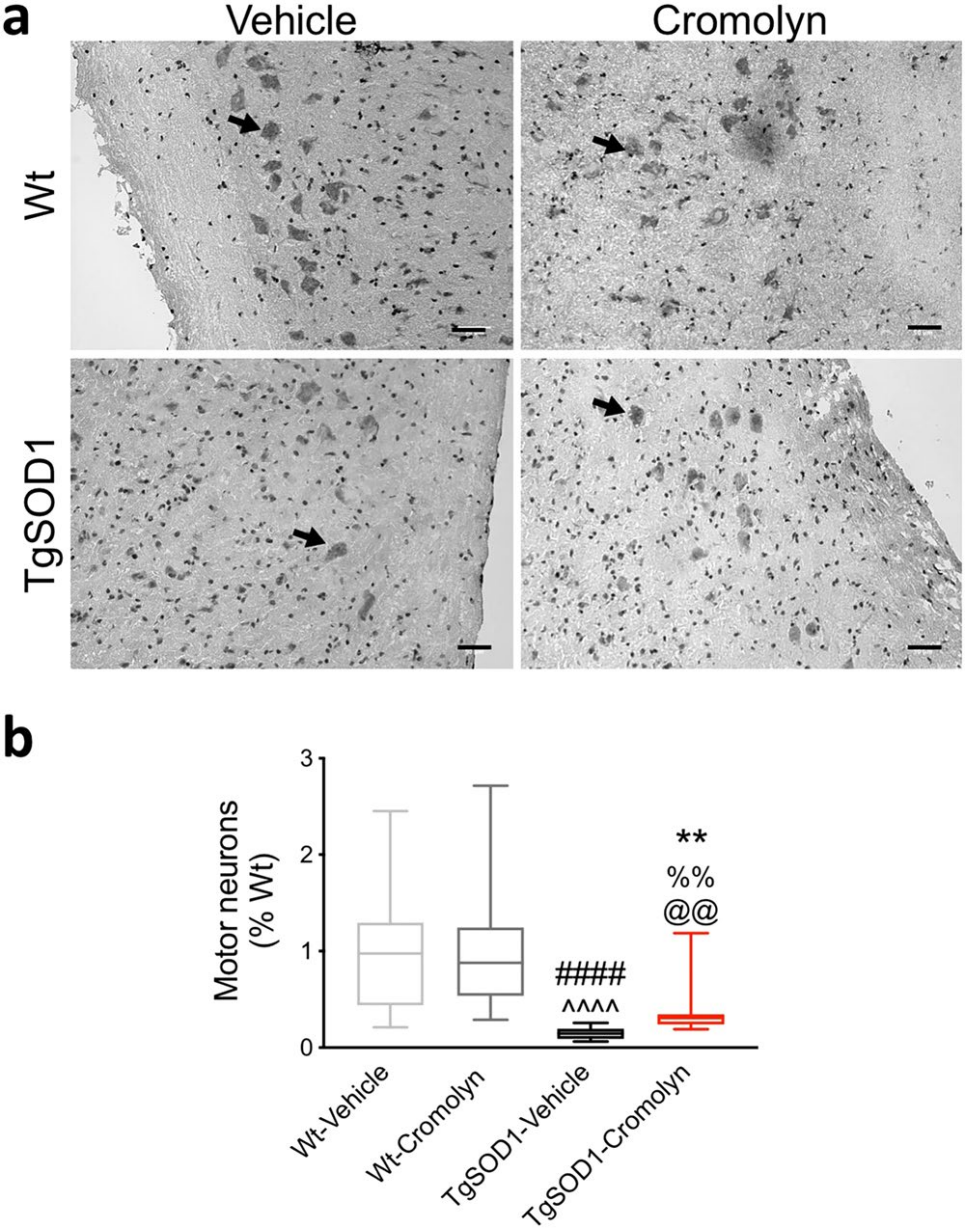
Cromolyn treatment is neuroprotective and increases survival of lumbar spinal cord motor neurons in TgSOD1 mice. (**a**) Representative images of lumbar spinal cord motor neurons visualized by H&E staining. (**b**) Two-way ANOVA and Dunn’s multiple comparisons test demonstrated that motor neuron survival was significantly increased in the TgSOD1-Cromolyn group compared to TgSOD1-Vehicle group. There was a significant decrease in motor neuron counts in the TgSOD1-Vehicle group compared to Wt-Vehicle and Wt-Cromolyn groups. There was also a decrease in motor neuron counts in TgSOD1-Cromolyn group compared to both Wt groups. Wt-Vehicle (n = 19; light grey), Wt-Cromolyn (n = 17; dark grey), TgSOD1-Vehicle (n = 19; black), and TgSOD1-Cromolyn (n = 17; red). *Denotes differences between TgSOD1-Vehicle and Tg-SOD1-Cromolyn; ^^^denotes differences between TgSOD1-Vehicle and Wt-Vehicle; ^#^denotes differences between TgSOD1-Vehicle and Wt-Cromolyn; ^@^denotes differences between TgSOD1-Cromolyn and Wt-Vehicle; ^%^denotes differences between TgSOD1-Cromolyn and Wt-Cromolyn. **p < 0.01; ****p < 0.0001, the same statistical significance is associated with each symbol. Data are presented as median and interquartile ranges. Scale bar = 50 μm.

Next, we assessed the effect of cromolyn treatment on lumbar spinal cord motor neuron counts. Motor neurons of the lumbar spinal cord were visualized using hematoxylin and eosin (H&E) staining (Fig. 4a) and a two-way ANOVA demonstrated a significant effect of genotype between the groups [F(1, 80) = 64.67, p < 0.0001]. Tukey’s multiple comparisons test demonstrated a significant decrease in motor neuron counts in the TgSOD1-Vehicle group compared to Wt-Vehicle (p < 0.0001) and Wt-Cromolyn (p < 0.0001) groups (Fig. 4b). Furthermore, there was a significant decrease in motor neuron counts between TgSOD1-Cromolyn and Wt-Vehicle (p = 0.0002) and Wt-Cromolyn (p = 0.0001) groups. Importantly, motor neuron survival was significantly increased in the TgSOD1-Cromolyn compared to TgSOD1-Vehicle group (p = 0.0458) (Fig. 4b), suggesting that cromolyn treatment is neuroprotective.

### Cromolyn treatment improved NMJ integrity in TgSOD1 mice

**Figure 5:**
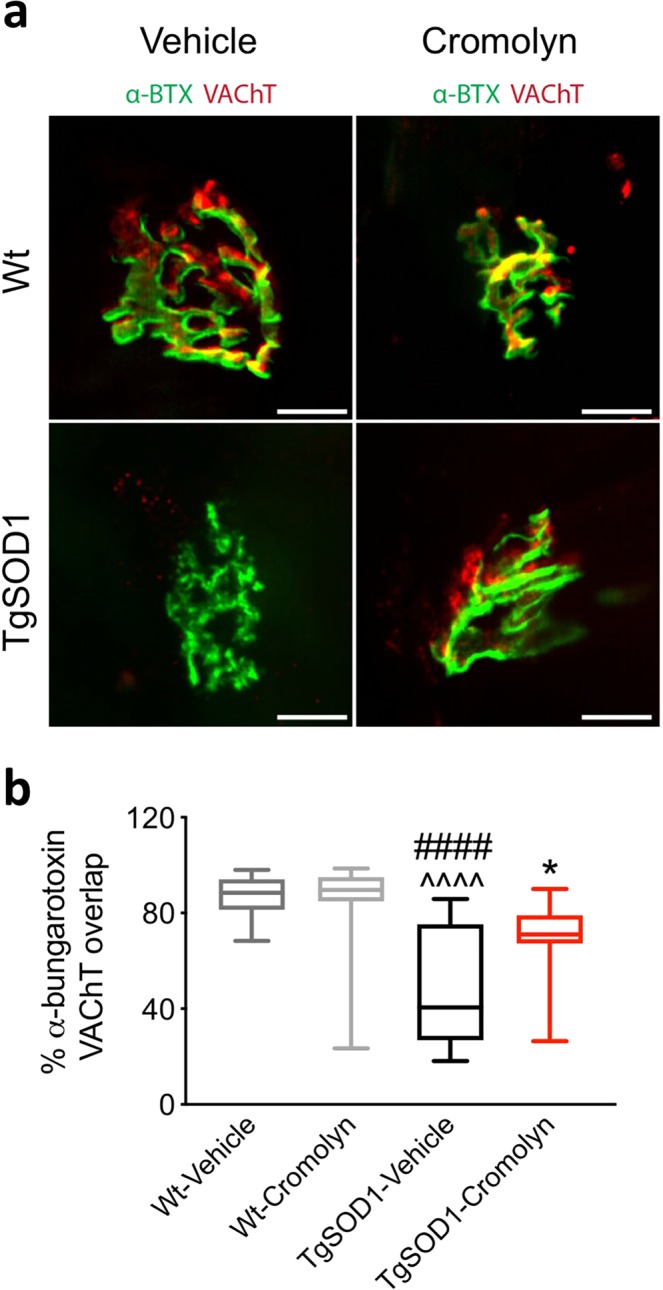
Cromolyn treatment decreases NMJ denervation of tibialis anterior muscle in TgSOD1 mice. (**a**) Representative NMJ of tibialis anterior muscle stained with α-BTX (green) and VAChT (red). (**b**) Quantification of the percent overlay between α-BTX and VAChT demonstrates a significant decrease in relative overlap in TgSOD1-Vehicle group compared to both Wt groups. There is also a significant increase in the overlap of α-BTX and VAChT in TgSOD1-Cromolyn group compared to TgSOD1-Vehicle. Wt-Vehicle (n = 13; light grey), Wt-Cromolyn (n = 13; dark grey), TgSOD1-Vehicle (n = 15; black), and TgSOD1-Cromolyn (n = 17; red). *Denotes differences between TgSOD1-Vehicle and Tg-SOD1-Cromolyn; ^^^denotes differences between TgSOD1-Vehicle and Wt-Vehicle; ^#^denotes differences between TgSOD1-Vehicle and Wt-Cromolyn; ^@^denotes differences between TgSOD1-Cromolyn and Wt-Vehicle; ^%^denotes differences between TgSOD1-Cromolyn and Wt-Cromolyn. *p < 0.05; ****p < 0.0001, the same statistical significance is associated with each symbol. Data are presented as median and interquartile ranges. Scale bar = 10 μm.

The effects of cromolyn treatment were assessed on NMJ integrity. Two-way ANOVA demonstrated a significant effect of genotype [F(1, 51) = 28.17, p < 0.0001] and a significant genotype X treatment interaction [F(1, 51) = 4.512, p < 0.0385]. In agreement with previous findings^14,15^, Tukey’s multiple comparisons test demonstrated a significant increase in denervated NMJs in TgSOD1-vehicle mice as indicated by a decrease in the overlap of presynaptic (vesicular acetylcholine transporter; VAChT) and postsynaptic (α-bungarotoxin; α-BTX) markers in the tibialis anterior muscle relative to Wt-Vehicle (p < 0.0001) and Wt-Cromolyn (p < 0.0001) (Fig. 5a,b). Although there was no significant difference between the TgSOD1-Cromolyn group with either Wt groups, there was a significant decrease in tibialis anterior NMJ denervation compared to the TgSOD1-Vehicle group (p = 0.0265) (Fig. 5b), suggesting that cromolyn treatment preserves NMJ innervation.

### Cromolyn treatment does not alter microgliosis or astrogliosis in the spinal cord of TgSOD1 mice

**Figure 6:**
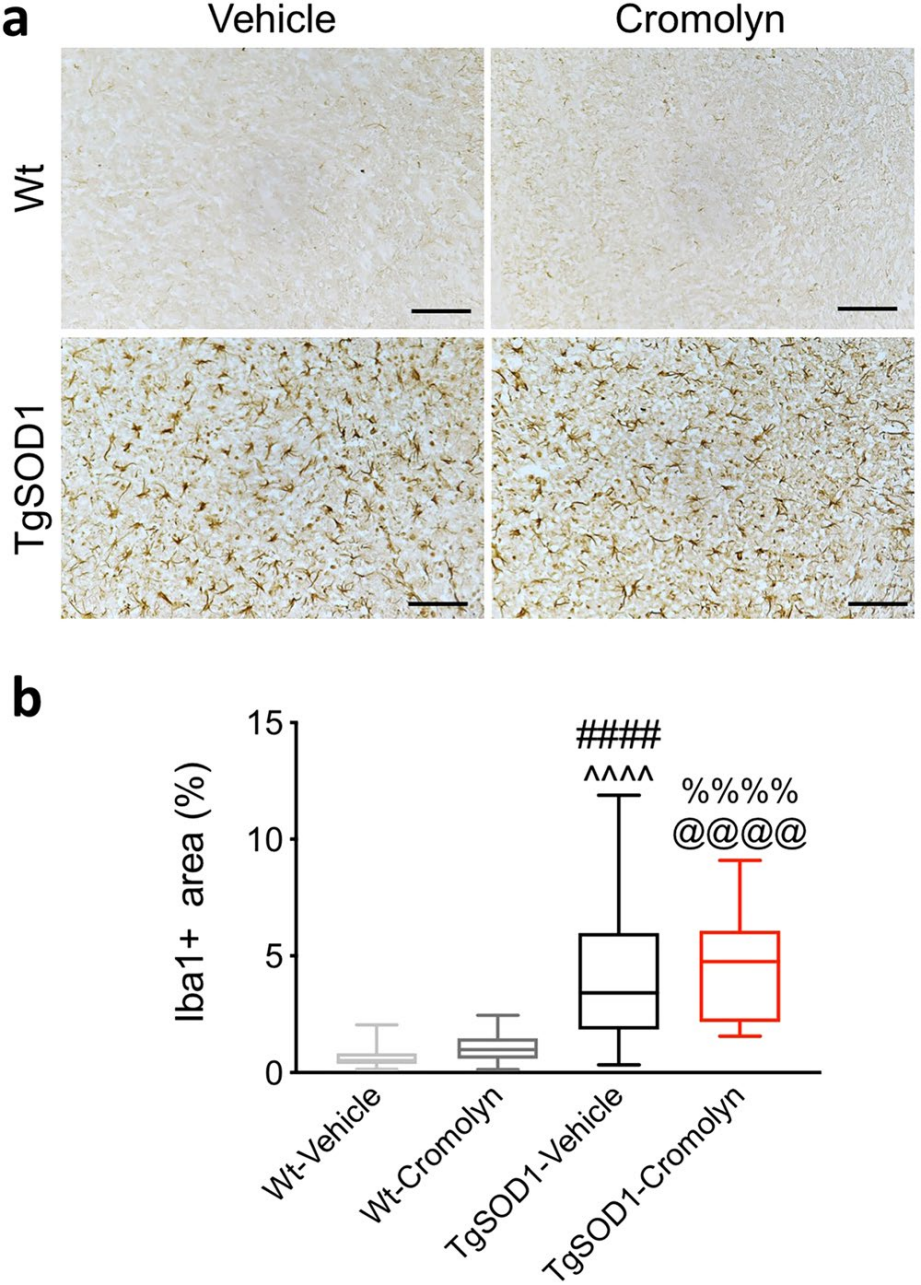
Cromolyn treatment does not alter microgliosis in the spinal cord of TgSOD1 mice. (**a**) Microglia of the lumbar spinal cord were visualized using the Iba1-specific antibody and DAB staining. (**b**) Quantifications of the percentage of Iba1^+^ cell area revealed no difference in the percentage of Iba1^+^ cell area in TgSOD1-Cromolyn mice compared to TgSOD1-Vehicle. There was a significant increase in the percentage of Iba1^+^ cell area in the spinal cord of both TgSOD1-Vehicle and TgSOD1-Cromolyn compared to both Wt groups as demonstrated by two-way ANOVA and Tukey’s post-hoc analysis. Wt-Vehicle (n = 19; light grey), Wt-Cromolyn (n = 17; dark grey), TgSOD1-Vehicle (n = 19; black), and TgSOD1-Cromolyn (n = 17; red). *Denotes differences between TgSOD1-Vehicle and Tg-SOD1-Cromolyn; ^^^denotes differences between TgSOD1-Vehicle and Wt-Vehicle; ^#^denotes differences between TgSOD1-Vehicle and Wt-Cromolyn; ^@^denotes differences between TgSOD1-Cromolyn and Wt-Vehicle; ^%^denotes differences between TgSOD1-Cromolyn and Wt-Cromolyn. ****p < 0.0001, the same statistical significance is associated with each symbol. Data are presented as median and interquartile ranges. Scale bar = 500 μm.

While acute treatment with cromolyn for one week was previously shown to lead to an increased number of microglia around β-amyloid plaques^9^, chronic treatment significantly promoted microglial uptake and clearance of Aβ^10^. Therefore, we assessed the effect of cromolyn treatment on microgliosis - by quantifying the percentage of microglial cells per lumbar spinal cord area. Microglial marker Iba1 was used to determine if similar effects could be observed after chronic treatment in the TgSOD1 mice. Two-way ANOVA demonstrated a significant effect of genotype [F(1, 78) = 60.03, p < 0.0001] as reported previously^16^. There was a significant increase in the percentage of Iba1^+^ cell area in the lumbar spinal cord of vehicle treated TgSOD1 mice compared to wild-type mice (Fig. 6a,b). In addition, there was a significant increase in the percentage of Iba1^+^ cell area in the spinal cord of both vehicle and cromolyn-treated TgSOD1 compared to both Wt groups as demonstrated by Tukey’s post-hoc analysis (p < 0.0001) (Fig. 6b). However, there was no significant change in the percentage of Iba1^+^ cell area in the lumbar spinal cord of TgSOD1-Cromolyn compared to TgSOD1-Vehicle (p < 0.982) (Fig. 6b). These data indicate that cromolyn treatment does not alter microgliosis in the lumbar spinal cord of TgSOD1 mice.

Next, we assessed the effect of cromolyn treatment on shifting microglia from pro-inflammatory to a pro-phagocytic state in a subset of spinal cord samples based on availability, using immunohistochemistry. Lumbar spinal cord sections were labeled with an antibody specific for CD68, which is a marker for phagocytosis shown to be upregulated in the ventral horn of spinal cord sections from ALS patients^17^. We quantified the percentage of CD68^+^ microglial cells per lumbar spinal cord area. Two-way ANOVA revealed a significant effect of genotype [F(1, 52) = 99.23, p < 0.0001] (Fig. S8). As reported previously^6^, Tukey’s post-hoc analysis revealed a significant increase in CD68 expression in the lumbar spinal cord of TgSOD1-Vehicle mice compared to Wt-Vehicle (p < 0.0001) and Wt-Cromolyn (p < 0.0001). Furthermore, there was a significant increase in CD68^+^ cell area in TgSOD1-Cromolyn group compared to Wt-Vehicle (p < 0.0001) and Wt-Cromolyn (p < 0.0001) groups. However, there was no significant difference in the percentage of CD68^+^ cell area between TgSOD1-Vehicle and TgSOD1-Cromolyn groups (p = 0.998), suggesting that cromolyn treatment does not alter CD68^+^ cell area (Fig. S8b).

We also assessed the effect of cromolyn treatment on astrogliosis in a subset of spinal cord samples based on availability by quantifying GFAP expression via immunofluorescence (Fig. S9). Two-way ANOVA demonstrated a significant effect of genotype [F(1, 48) = 10.95, p < 0.0018] on astrogliosis in the spinal cord. In addition, Tukey’s post-hoc analysis revealed a significant increase in GFAP^+^ staining in the TgSOD1-Cromolyn group compared to Wt-Vehicle (p = 0.0028) and Wt-Cromolyn (p = 0.0373) groups in the spinal cord (Fig. S9b), as reported previously^18^. However, there was no significant difference in GFAP^+^ cell area between TgSOD1-Cromolyn and TgSOD1-Vehicle groups (p = 0.923), suggesting that cromolyn treatment does not impact astrogliosis in the TgSOD1 mouse model of ALS.

### Cromolyn treatment decreased the levels of pro-inflammatory cytokines/chemokines in the spinal cord of TgSOD1 mice

**Figure 7:**
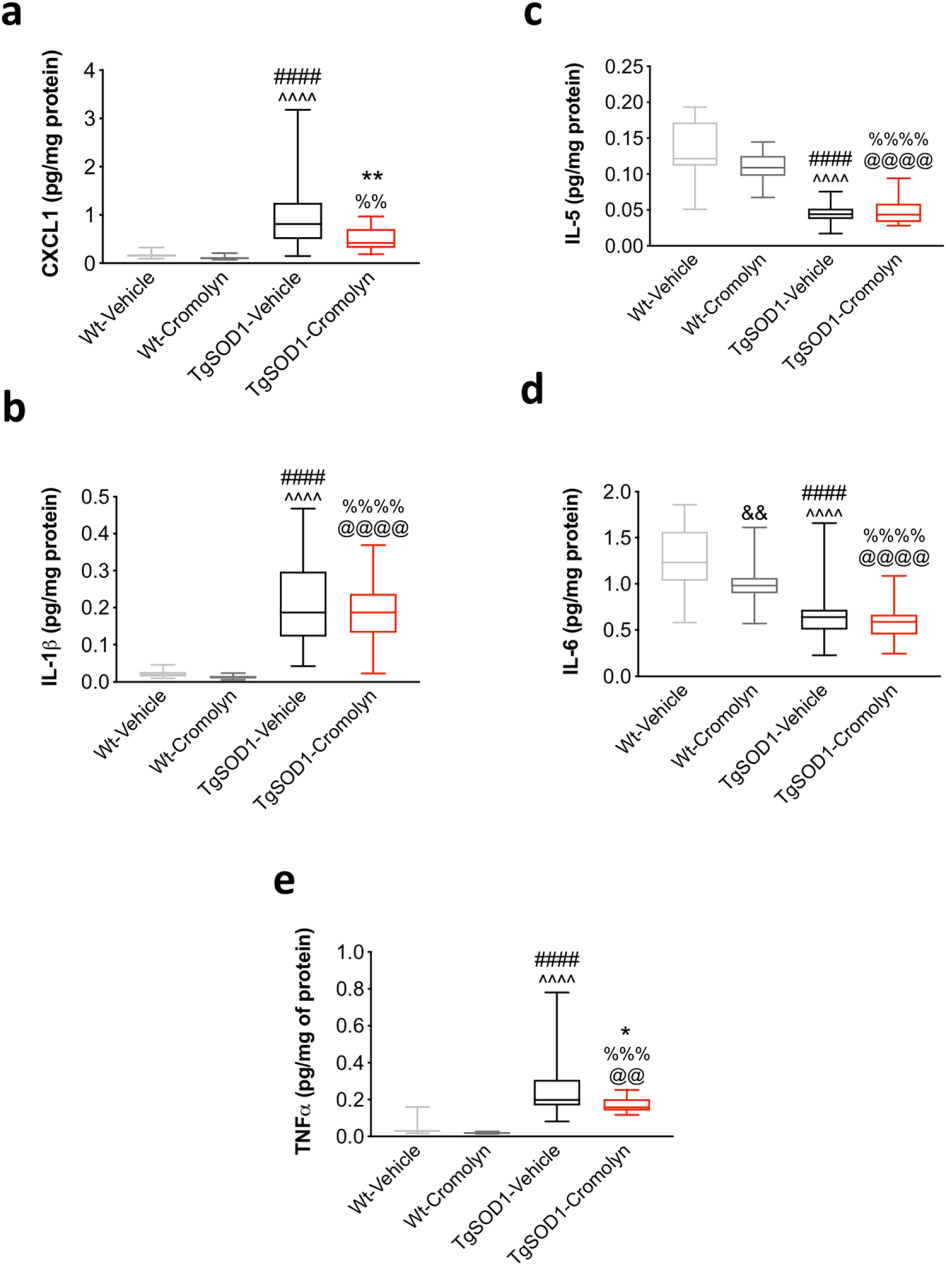
Cromolyn treatment decreased the levels of pro-inflammatory cytokines/chemokines in the spinal cord of TgSOD1 mice. Two-way ANOVA and post-hoc analysis revealed that cromolyn treatment significantly decreased CXCL1 (**a**) and TNFα (**e**) levels in the TgSOD1-Cromolyn group compared to TgSOD1-Vehicle group. There was a significant difference in the levels of CXCL1 (**a**), IL-1β (**b**), IL-5 (**c**), IL-6 (**d**), and TNFα (**e**) in the spinal cord of both TgSOD1-Vehicle and TgSOD1-Cromolyn groups compared to both Wt groups. While there was a significant increase in CXCL1 (**a**), IL-1β (**a**), and TNFα (**e**), there was a significant decrease in IL-5 (**c**) and IL-6 (**d**) levels between Tg and Wt groups. Wt-Vehicle (n = 15; light grey), Wt-Cromolyn (n = 19; dark grey), TgSOD1-Vehicle (n = 17; black), and TgSOD1-Cromolyn (n = 17; red). *Denotes differences between TgSOD1-Vehicle and Tg-SOD1-Cromolyn; ^ denotes differences between TgSOD1-Vehicle and Wt-Vehicle; ^#^denotes differences between TgSOD1-Vehicle and Wt-Cromolyn; ^@^denotes differences between TgSOD1-Cromolyn and Wt-Vehicle; ^%^denotes differences between TgSOD1-Cromolyn and Wt-Cromolyn. *p < 0.05; **p < 0.01; ***p < 0.001; ****p < 0.0001, the same statistical significance is associated with each symbol. Data are presented as median and interquartile ranges.

To assess the effects of cromolyn treatment on inflammation, we measured the levels of pro-inflammatory cytokines and chemokines in spinal cord lysates of mice by using the multi-spot assay system from Meso Scale Discovery (Fig. 7). This assay allows for the simultaneous measurement of 10 cytokines and chemokines including: IFN-γ, IL-1β, IL-2, IL-4, IL-5, IL-6, CXCL1, IL-10, IL-12, and TNFα, which are known to be important in the neuroinflammatory response. Of these 10 cytokines and chemokines, we were able to reliably detect only 5 including IL-1β, IL-5, IL-6, CXCL1, and TNFα. Two-way ANOVA revealed a significant effect of genotype [F(1, 63) = 35.61, p < 0.0001], treatment [F(1, 63) = 5.428, p < 0.023] and genotype X treatment interaction [F(1, 63) = 4.787, p < 0.0324] on CXCL1 levels in the spinal cord. Tukey’s post-hoc analysis revealed a significant increase in CXCL1 in the TgSOD1-Vehicle group compared to Wt-Vehicle (p < 0.0001) and Wt-Cromolyn (p < 0.0001). In addition, there was a significant increase in CXCL1 levels in TgSOD1-Cromolyn group compared Wt-Cromolyn (p = 0.0349). Importantly there was a significant decrease in CXCL1 levels in TgSOD1-Cromolyn compared to TgSOD1-Vehicle (p = 0.0101) (Fig. 7a). Two-way ANOVA also revealed a significant effect of genotype on IL-1β [F(1, 132) = 192.3, p < 0.0001] in the spinal cord. Tukey’s post-hoc analysis revealed a significant increase in IL-1β in both TgSOD1 groups compared to Wt-Vehicle (p < 0.0001) and Wt-Cromolyn (p < 0.0001) (Fig. 7b). Two-way ANOVA revealed a significant effect of genotype [F(1, 129) = 378.3, p < 0.0001], treatment [F(1, 129) = 4.236, p < 0.0416], and genotype X treatment interaction [F(1, 129) = 7.240, p < 0.0081] on IL-5 levels in the spinal cord. In addition, Tukey’s post-hoc analysis revealed a significant decrease in IL-5 in both TgSOD1 groups compared to both Wt groups (p < 0.0001) (Fig. 7c). Two-way ANOVA revealed a significant effect of genotype [F(1, 133) = 108.3, p < 0.0001] and treatment [F(1, 133) = 16.83, p < 0.0001] on IL-6 levels in the spinal cord. In addition, Tukey’s post-hoc analysis revealed a significant decrease in IL-6 in both TgSOD1 groups compared to both Wt groups (p < 0.0001) (Fig. 7d). Lastly, Two-way ANOVA revealed a significant effect of genotype [F(1, 63) = 70.7, p < 0.0001] and treatment [F(1, 63) = 6.123, p = 0.0161] on TNFα levels in the spinal cord. In addition, Tukey’s post-hoc analysis revealed a significant increase in TNFα in the TgSOD1-Vehicle group compared to both Wt groups (p < 0.0001), while there was also a significant increase in TNFα in the Tg-SOD1-Cromolyn group compared to Wt-Vehicle (p = 0.0007) and Wt-Cromolyn (p < 0.0001). Importantly, there was a significant decrease in TNFα in the TgSOD1-Cromolyn group compared to TgSOD1-Vehicle (p = 0.0218) in the spinal cord (Fig. 7e). Together these findings suggest that cromolyn treatment decreased the expression of pro-inflammatory cytokines and chemokines in the spinal cord of treated transgenic mice.

### Cromolyn treatment decreased the levels of pro-inflammatory cytokines/chemokines in plasma of TgSOD1 mice

**Figure 8:**
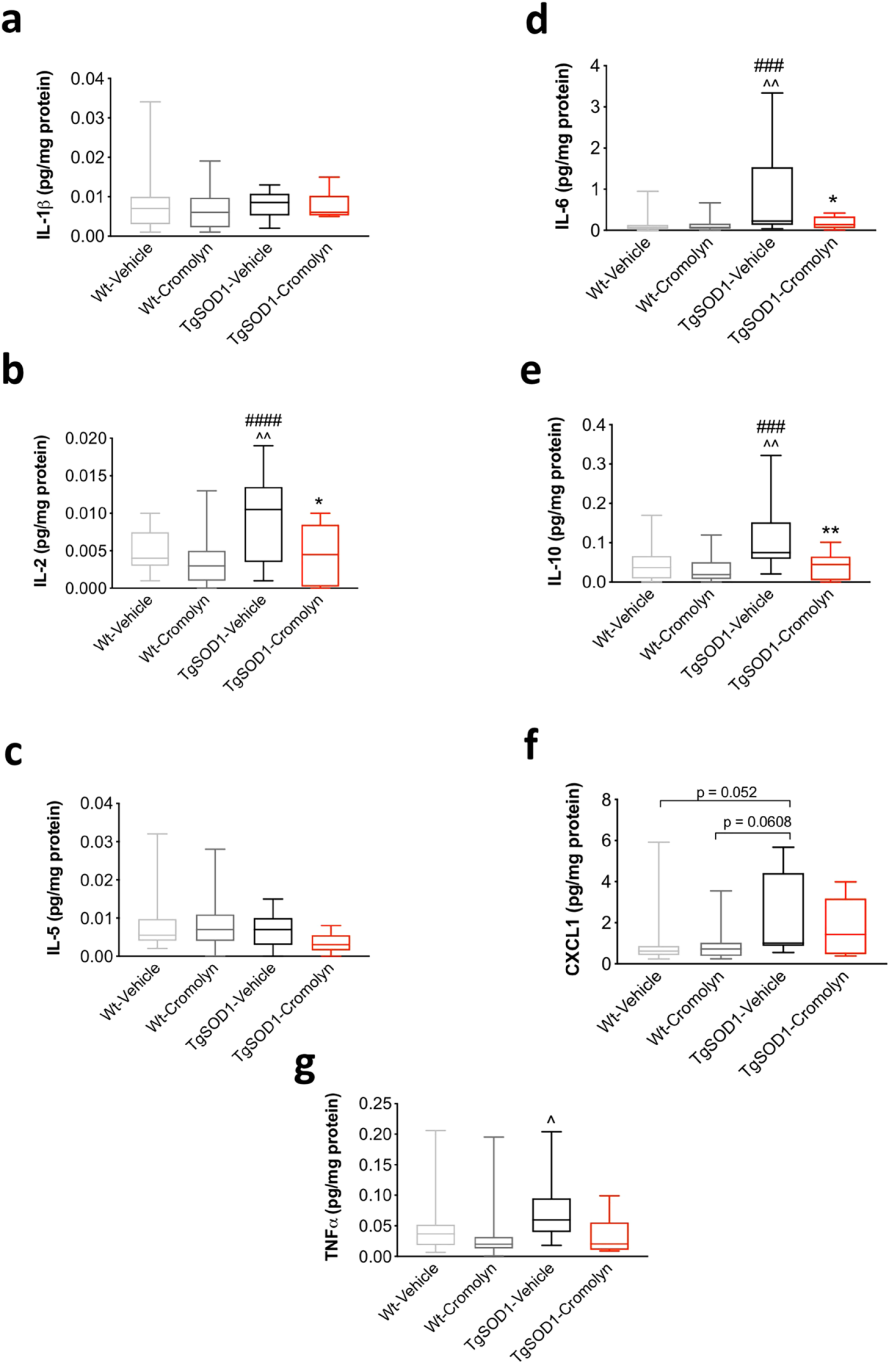
Cromolyn treatment decreased the levels of pro-inflammatory cytokines/chemokines in plasma of TgSOD1 mice. (**a**) Two-way ANOVA and post-hoc analysis revealed a significant decrease in IL-2 (**b**), IL-6 (**d**), and IL-10 (**e**) levels in TgSOD1-Cromolyn group compared to TgSOD1-Vehicle group. There was a significant difference in IL-2 (**b**), IL-6 (**d**), and IL-10 (**e**) levels in the plasma of TgSOD1-Vehicle compared to both Wt-Vehicle and Wt-Cromolyn groups. Two-way ANOVA demonstrated a trend towards an increase in CXCL1 (**f**) levels in TgSOD1-Vehicle mice compared to Wt-Vehicle group and a trend towards an increase compared to Wt-Cromolyn group. There was no statistically significant difference between IL-1β (**a**) and IL-5 (**c**) levels between groups. Lastly, there was a trend towards a decrease in TNFα levels (**g**) in the TgSOD1-Cromolyn mice compared to the TgSOD1-Vehicle group. Wt-Vehicle (n = 11; light grey), Wt-Cromolyn (n = 11; dark grey), TgSOD1-Vehicle (n = 9; black), and TgSOD1-Cromolyn (n = 9; red). *Denotes differences between TgSOD1-Vehicle and Tg-SOD1-Cromolyn; ^ denotes differences between TgSOD1-Vehicle and Wt-Vehicle; ^#^denotes differences between TgSOD1-Vehicle and Wt-Cromolyn; ^@^denotes differences between TgSOD1-Cromolyn and Wt-Vehicle; ^%^denotes differences between TgSOD1-Cromolyn and Wt-Cromolyn. *p < 0.05; **p < 0.01; ***p < 0.001; ****p < 0.0001, the same statistical significance is associated with each symbol. Data are presented as median and interquartile ranges.

The same pro-inflammatory panel from Meso Scale Discovery was used to assess the levels of cytokines and chemokines in the plasma of a subset of mice (Females: 13 Wt-Vehicle, 15 Wt-Cromolyn, 6 TgSOD1-Vehicle, and 6 TgSOD1-Cromolyn; and Males: 14 Wt-Vehicle, 10 Wt-Cromolyn, 6 TgSOD1-Vehicle, 3 TgSOD1-Cromolyn). We were able to measure 7 of the 10 cytokines and chemokines in plasma which included IL-1β, IL-2, IL-5, IL-6, CXCL1, IL-10, and TNFα. Two-way ANOVA revealed that there was no significant effect of treatment on IL-1β (Fig. 8a) or IL-5 (Fig. 8c) levels. However, there was a significant effect of genotype [F(1, 63) = 7.272, p = 0.009] and treatment [F(1, 63) = 11.34, p = 0.0013] on plasma IL-2 levels. Tukey’s post-hoc analysis revealed a significant increase in IL-2 in the TgSOD1-Vehicle group compared to Wt-Vehicle (p = 0.009) and Wt-Cromolyn (p = 0.0001). Importantly, there was a significant decrease in plasma IL-2 levels in the TgSOD1-Cromolyn group compared to TgSOD1-Vehicle (p = 0.0234) (Fig. 8b). Two-way ANOVA also revealed a significant effect of genotype [F(1, 63) = 8.168, p = 0.0058], treatment [F(1, 63) = 6.249, p = 0.015] and genotype X treatment interaction [F(1, 63) = 5.697, p = 0.02] on plasma IL-6 levels. Moreover, Tukey’s post-hoc analysis revealed a significant increase in IL-6 in the TgSOD1-Vehicle group compared to Wt-Vehicle (p = 0.0011) and Wt-Cromolyn (p = 0.001). Importantly, there was a significant decrease in plasma IL-6 levels in the TgSOD1-Cromolyn group compared to TgSOD1-Vehicle (p = 0.0273) (Fig. 8d). Two-way ANOVA also revealed a significant effect of genotype [F(1, 65) = 7.907, p = 0.0065], treatment [F(1, 65) = 11.72, p = 0.0011] and genotype X treatment interaction [F(1, 65) = 4.471, p = 0.038] on plasma IL-10 levels. Moreover, Tukey’s post-hoc analysis revealed a significant increase in IL-10 in the TgSOD1-Vehicle group compared to Wt-Vehicle (p = 0.0031) and Wt-Cromolyn (p = 0.0001). Importantly, there was a significant decrease in plasma IL-10 levels in the TgSOD1-Cromolyn group compared to TgSOD1-Vehicle (p = 0.0088) (Fig. 8e). Two-way ANOVA revealed a significant effect of genotype [F(1, 65) = 8.508, p = 0.0048] on plasma CXCL1 levels. In addition, Tukey’s post-hoc analysis revealed a trend towards a significant increase in CXCL1 in the TgSOD1-Vehicle group compared to Wt-Vehicle (p = 0.052) (Fig. 8f). Lastly, Two-way ANOVA revealed a significant effect of treatment [F(1, 63) = 6.682, p = 0.0121] on plasma TNFα levels. In addition, Tukey’s post-hoc analysis revealed a significant increase in TNFα in the TgSOD1-Vehicle group compared to Wt-Cromolyn (p = 0.0355) (Fig. 8g). Together, these findings demonstrate a significant effect of cromolyn treatment on IL-2, IL-6, and IL-10 levels in plasma of treated Tg mice.

Previous studies have demonstrated that CCL2/MCP-1 levels are significantly increased in ALS^19–21^. We were however unable to measure MCP-1 levels using the Meso Scale Discovery assay, as it was not represented on the specific panel that we used for our analysis. Therefore, we assessed the effect of cromolyn treatment on MCP-1 levels using an ELISA assay in the same spinal cord and plasma samples. One-way ANOVA and Tukey’s post-hoc analysis revealed that the levels of MCP-1 were significantly increased in the spinal cord of TgSOD1-Cromolyn mice compared to both Wt-Vehicle and Wt-Cromolyn groups [F(3, 92) = 46.24, p < 0.0001] (Fig. S10a). However, there was no effect of cromolyn treatment on MCP-1 levels in the spinal cord of TgSOD1-Cromolyn compared to TgSOD1-Vehicle. Moreover, MCP-1 levels were not altered in the plasma in any of the groups [F(3, 32) = 2.357, p < 0.0902] (Fig. S10b). Thus, cromolyn treatment had no effect on MCP-1 levels in spinal cord or plasma of TgSOD1 mice.

### Cromolyn treatment decreases mast cell degranulation in the anterior tibialis muscle TgSOD1 mice

**Figure 9:**
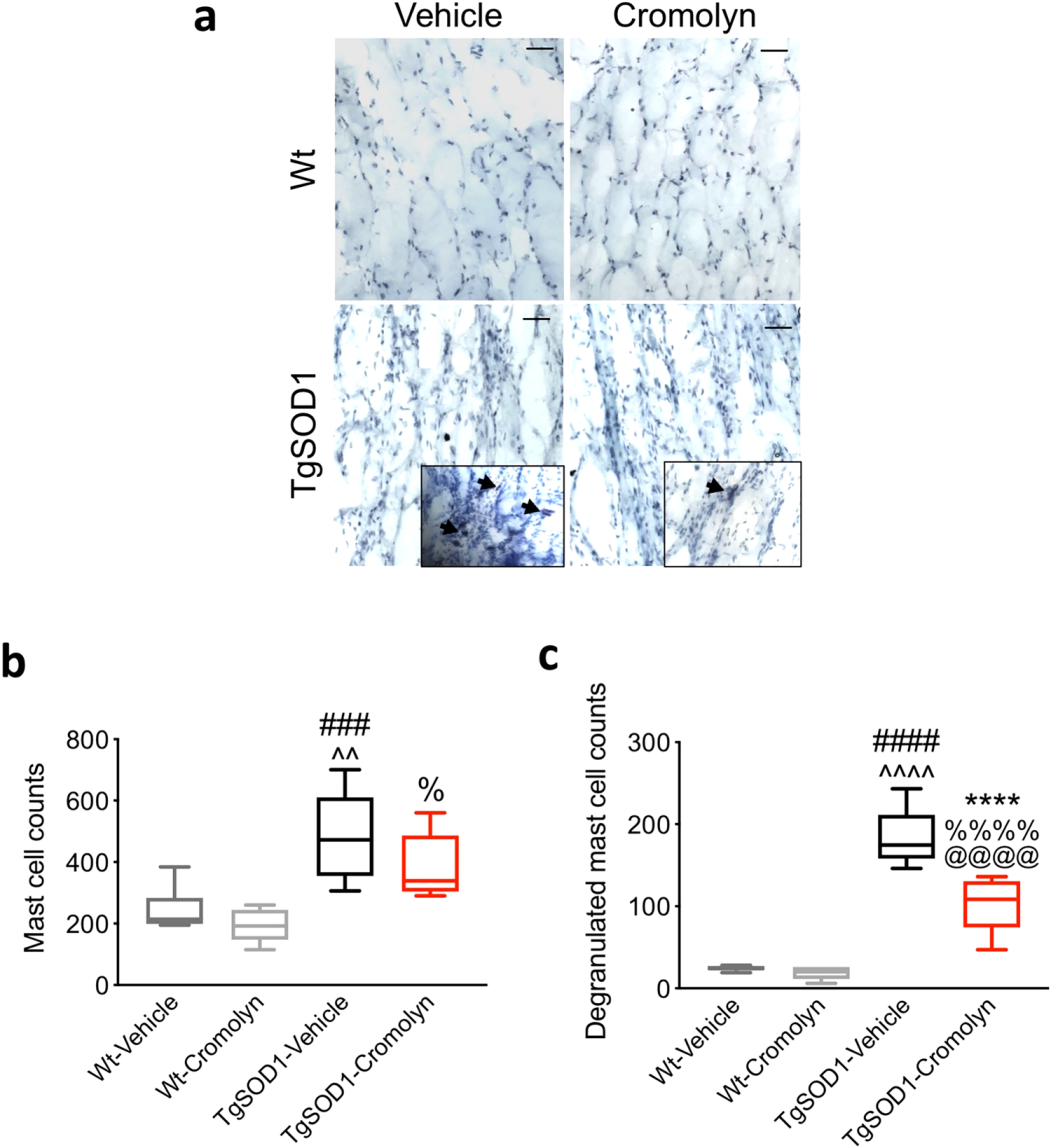
Cromolyn treatment decreases mast cell degranulation in the tibialis anterior muscle. (**a**) Mast cells of the tibialis anterior muscle were visualized by toluidine blue staining. (**b**) Quantification of the number of mast cells revealed no difference in the total numbers in TgSOD1-Cromolyn mice compared to TgSOD1-Vehicle. There was a significant increase in the number of mast cells in TgSOD1-Vehicle compared to both Wt groups as demonstrated by two-way ANOVA and Tukey’s post-hoc analysis. There was also an increase in the number of mast cells in the TgSOD1-Cromolyn group compared to Wt-Cromolyn group. (**c**) Quantification of the number of degranulated mast cells revealed a significant decrease in the TgSOD1-Cromolyn mice compared to TgSOD1-Vehicle. There was a significant increase in the number of degranulated mast cells in both TgSOD1-Vehicle and TgSOD1-Cromolyn compared to both Wt groups as demonstrated by two-way ANOVA and Tukey’s post-hoc analysis. Wt-Vehicle (n = 7; light grey), Wt-Cromolyn (n = 5; dark grey), TgSOD1-Vehicle (n = 9; black), and TgSOD1-Cromolyn (n = 9; red). *Denotes differences between TgSOD1-Vehicle and Tg-SOD1-Cromolyn; ^^^denotes differences between TgSOD1-Vehicle and Wt-Vehicle; ^#^denotes differences between TgSOD1-Vehicle and Wt-Cromolyn; ^@^denotes differences between TgSOD1-Cromolyn and Wt-Vehicle; ^%^denotes differences between TgSOD1-Cromolyn and Wt-Cromolyn. *p < 0.05; **p < 0.01; ***p < 0.001; ****p < 0.0001, the same statistical significance is associated with each symbol. Data are presented as median and interquartile ranges. Scale bar = 50 μm.

Cromolyn has been shown to inhibit mast cell activation and degranulation^22,23^. Therefore, we next assessed the effects of cromolyn treatment on mast cell number and degranulation in the tibialis anterior muscle (Fig. 9). Two-way ANOVA demonstrated a significant effect of genotype [F(1, 23) = 27.83, p < 0.0001] on the number of mast cells in the tibialis anterior muscle (Fig. 9b). Furthermore, Tukey’s post-hoc analysis revealed a significant increase in the number of mast cells in the TgSOD1-Vehicle group compared to Wt-Vehicle (p = 0.0016) and Wt-Cromolyn (p = 0.0004). There was also a significant increase in the number of mast cells in the TgSOD1-Cromolyn group compared to Wt-Cromolyn (p = 0.0173) (Fig. 9b). However, there was no significant difference in the number of mast cells between the two Tg groups (Fig. 9b). Next, we measured the number of degranulated mast cells between groups following treatment. Two-way ANOVA demonstrated that there was significant effect of genotype [F(1, 24) = 159.9, p < 0.0001], treatment [F(1, 24) = 20.64, p = 0.0001], and genotype X treatment interaction [F(1, 24) = 15.63, p = 0.0006] in the number of degranulated mast cells between groups (Fig. 9c). Tukey’s post-hoc analysis revealed a significant increase in the number of degranulated mast cells in the TgSOD1-Vehicle group compared to Wt-Vehicle (p < 0.0001) and Wt-Cromolyn (p < 0.0001). There was also a significant increase in the number of degranulated mast cells in the TgSOD1-Cromolyn group compared to both Wt groups (p < 0.0001) (Fig. 9b). Importantly, there was a significant decrease in the number of degranulated mast cells in the tibialis anterior in the TgSOD1-Cromolyn group compared to TgSOD1-Vehicle group (p < 0.0001). Together, these findings suggest that cromolyn treatment stabilizes mast cells and decreases degranulation in the tibialis anterior muscle.

## Discussion

Our results demonstrate that cromolyn sodium treatment delayed disease onset and reduced motor deficits in the PaGE task in the SOD1^*G93A*^ mouse model. Furthermore, cromolyn treatment significantly spared lumbar spinal cord motor neurons and reduced denervation at the NMJ in tibialis anterior muscle in treated TgSOD1 mice. Lastly, cromolyn treatment decreased pro-inflammatory cytokine/chemokine levels in the spinal cord and plasma of TgSOD1 mice and significantly reduced the number of degranulated mast cells in the tibialis anterior. Together, these findings suggest that cromolyn may provide neuroprotective effects by regulating the immune response in TgSOD1 mice.

Cromolyn inhibits mast cell degranulation and is used to treat asthma, allergic rhinitis, mastocytosis, and conjunctivitis^22,23^. In animal models, cromolyn treatment attenuates activation and degranulation of mast cells, reduces histamine expression and infiltration of macrophages^23^. Furthermore, cromolyn decreases the expression of pro-inflammatory chemokines and cytokines such as IL-1β, IL-6, TNFα, CCL3 and MCP1^23,24^. Previously, we had demonstrated that cromolyn decreases Aβ aggregation *in vitro* and reduces the levels of soluble monomeric Aβ in the transgenic APPswe/PS1σE9 mouse model of AD^9^. Recently, we reported that cromolyn significantly reduced insoluble Aβ levels in the brain of APP^Swedish^-expressing Tg2576 mice^10^. Both of these studies suggested that the neuroprotective effects of cromolyn may be due to an increased recruitment of microglia to amyloid plaques, as cromolyn was also shown to upregulate microglial uptake of Aβ in a dose-dependent manner in a microglial cell-based assay. These observed effects of cromolyn suggest that treatment may convert microglial activation state from one favoring neuroinflammation to one promoting phagocytosis.

Here, we report that although microgliosis and astrogliosis were significantly increased in the spinal cord of TgSOD1 mice as shown previously^16,25^, there was no difference between vehicle and cromolyn treated TgSOD1 mice. Cromolyn treatment had no effect on the total number of astrocytes (GFAP), microglia (Iba1), or phagocytic microglia (CD68). We predict that CD68^+^ microglia provide a protective function during early stages of disease by clearing injured cells or debris in the spinal cord to prevent neuronal cell loss. Although, it is not clear whether phagocytic CD68^+^ microglial cells may become injurious during the late stages of disease when generalized inflammation is present^6^. Thus, our findings only demonstrate that cromolyn treatment does not impact CD68 expression in the lumbar spinal cord of TgSOD1 mice.

To further delineate the effects of cromolyn treatment on inflammation, we measured alterations in cytokines and chemokines in the spinal cord and plasma. Our findings demonstrate that pro-inflammatory cytokines, IL-1β and TNFα, and the chemokine CXCL1, were significantly increased in the spinal cord, while the cytokines IL-5 and IL-6 were decreased in the spinal cord of TgSOD1 mice compared to Wt mice, in agreement with previous reports^20^. Importantly, cromolyn sodium treatment significantly decreased CXCL1 and TNFα levels in the spinal cord of TgSOD1 mice in comparison to vehicle treatment. We also found that cromolyn treatment reduced the levels of IL-2, IL-6 and IL-10 in the plasma of TgSOD1 mice. The significant decrease in the levels of pro-inflammatory cytokines and chemokines in the spinal cord and plasma in response to cromolyn treatment may induce a shift in microglial activation states from pro- to anti-inflammatory. These results are in agreement with our previous study demonstrating that cromolyn promoted a neuroprotective microglial activation state favoring Aβ clearance versus a pro-inflammatory state^10^.

Although cromolyn treatment had no effect on the total number/cell area of microglia (Iba1) and astrocytes (GFAP), it is important to note that these cell numbers do not reflect the activation state of glial cells.

Liddelow *et al*.^25^ demonstrated that pro-inflammatory microglia induce neurotoxic reactive (A1) astrocytes by secreting IL-1α, TNF and C1q, and that these cytokines together are necessary and sufficient to induce reactive astrocytes. Neurotoxic reactive astrocytes promote the death of neurons and oligodendrocytes in the CNS. Given that our results demonstrated a significant decrease in pro-inflammatory cytokines/chemokines in the spinal cord and plasma of TgSOD1 mice following cromolyn treatment, they suggest that there may be a shift from a toxic/pro-inflammatory microglial activation state to an anti-inflammatory state, which subsequently could prevent the transition of astrocytes to a neurotoxic state.

CXCL1 is a chemotactic cytokine responsible for mediating migration of neutrophils to the site of inflammation^26–29^ and previous studies have demonstrated a significant increase in CXCL1 levels in ALS^29,30^. Specifically, CXCL1 levels were increased in monocytes isolated from ALS patients^29^ and in ALS patient-derived fibroblasts^30^. Here, we demonstrate that CXCL1 levels are increased in the spinal cord of TgSOD1 mice similar to previous reports in ALS patients, however, its role in ALS pathogenesis is yet unclear. Given that CXCL1 has been shown to contribute to the transendothelial migration of monocytes from blood to the brain in AD patients^24^, it may contribute to peripheral nerve invasion by macrophages in ALS patients. Therefore, lowering CXCL1 expression by using cromolyn could be highly beneficial for decreasing the inflammatory response in ALS patients.

Cromolyn sodium treatment also significantly decreased the levels of TNFα in the spinal cord of TgSOD1 mice. Although astrocytes and neurons are able to produce TNFα, it is assumed that microglia are the major source of TNFα release during neuroinflammation^31^. TNFα has been shown to potentiate AMPAR-mediated excitotoxicity on lumbar spinal cord motor neurons by decreasing GLT-1 expression^32^, and by inducing a rapid membrane insertion of Ca^2+^ permeable-AMPARs^33^. Therefore, cromolyn treatment could provide some of its neuroprotective effects by decreasing AMPA-mediated excitotoxicity.

Pro-inflammatory cytokines, such as IL-1β, TNF-α, IFN-γ, IL-6, and IL-8 are elevated in plasma or serum samples of ALS patients, with levels increasing with disease progression^29,34^. Furthermore, peripheral blood inflammatory cytokines have been suggested as diagnostic biomarkers for ALS^34^. Therefore, we also assessed the effects of cromolyn sodium treatment in the periphery to identify a pharmacodynamic biomarker for the treatment. Our results indicated that the cytokines IL-2, IL-6, IL-10, were increased in the plasma of TgSOD1 mice compared to Wt mice. Furthermore, we observed a significant increase in TNFα levels and a trend towards increase in CXCL1 levels in the plasma of TgSOD1 mice compared to Wt mice, similar to the findings in the spinal cord. Cromolyn treatment resulted in significantly decreased levels of IL-2, IL-6, and IL-10, as well as a trend towards decreased TNFα levels in the plasma, suggesting that cromolyn treatment reduces inflammation in the blood of TgSOD1 mice. The finding that cromolyn alters the levels of peripheral blood inflammatory cytokines is relevant for future investigations into the effects of cromolyn in people with ALS, as these cytokines may serve as biomarkers of target engagement. While IL-2 and TNFα are considered to be pro-inflammatory cytokines, IL-6 exhibits both pro- and anti-inflammatory properties^35^. Interestingly, IL-10 has been shown to inhibit inflammatory response by metabolic reprogramming of macrophages^36^. Given that peripheral blood inflammatory cytokines may provide novel diagnostic biomarkers for ALS^34^, it is critical that cromolyn treatment led to a decrease in the levels of these potential biomarkers. Although MCP-1 levels were increased in the spinal cord but not plasma of TgSOD1 mice (this study) and in the cerebrospinal fluid of ALS patients^19,20^, cromolyn treatment did not impact MCP-1 levels in the spinal cord or plasma of TgSOD1 mice.

Lastly, our results demonstrate a concomitant decrease in denervated NMJs in the tibialis anterior muscle together with a significant decrease in the number of degranulating mast cells following cromolyn treatment in the TgSOD1 mice. A previously published report demonstrated that a significant increase in mast cell numbers and degranulation in the SOD1^*G93A*^ rat correlated with paralysis progression^37^. Importantly, there was a significant decrease in mast cells, NMJ denervation, and motor deficits following treatment with masitinib, a tyrosine kinase inhibitor^37^, currently in clinical trials for the treatment of ALS (NCT03127267). Mast cells can accumulate in the muscle following injury^38^ and in ALS patients, mast cells are increased in the motor cortex and other regions of neurodegeneration^39^. Together, these findings suggest that accumulation and activation of mast cells at the NMJ can further accelerate denervation and therapies such as cromolyn that stabilize and decrease mast cell degranulation can protect the NMJ from further denervation.

Together, these findings demonstrate that cromolyn sodium treatment delays disease onset and reduces motor deficits (PaGE) in the SOD1^*G93A*^ mouse model. There was also a significant effect of cromolyn treatment on survival but only in the female mice. Furthermore, cromolyn treatment significantly increased the survival of lumbar spinal cord motor neurons and decreased denervation at the NMJ. While cromolyn treatment did not impact astrogliosis and microgliosis, it reduced pro-inflammatory cytokine and chemokine levels in the spinal cord and plasma of SOD1^*G93A*^ mice, and significantly decreased mast cell degranulation, suggesting that cromolyn alters inflammatory responses. Taken together, these findings provide the rationale for further investigating cromolyn as a possible therapeutic approach to dampen inflammation for the treatment of ALS.

## Materials and Methods

### Chemicals

Cromolyn sodium was provide by AZTherapies and dissolved in PBS. 100 mM solution was used for *in vivo* experiments. Dulbecco’s PBS was used to dilute the solution for intraperitoneal injections for a final dose of 6.3 mg/kg as described previously^9^.

### Animals

149 mice were used in this study. All animal care, husbandry and experimentation were performed according to the guidelines set by the Massachusetts General Hospital Subcommittee on Research Animal Care. These experiments were approved by the Massachusetts General Hospital Institutional Animal Care and Use Committee (2014N000018). All mice were given access to food and water ad libitum. Mice were assessed regularly for motor impairment and euthanized upon onset of major paralysis (neurological scoring = 4, see Neurological Score below) to minimize suffering as described previously^13^. Mice that exhibited mild paralysis (neurological score = 2) were given water bottles with long sipper tubes and hydrogel. The endpoint used in this study was based on previously reported criteria by ALS TDI: the loss of self-righting ability within 10 seconds (neurological score = 4) or the inability to move to reach food on the cage floor. Mice that reached the humane endpoint were euthanized within 3 hours.

#### SOD1^G93A^ mice

B6SJL-Tg (SOD1 G93A)1Gur/J transgenic male mice^40^ were obtained from Jackson Laboratory and bred with C57BL/6 female mice to obtain wild-type (Wt) SOD1 and mutant transgenic (Tg) SOD1^*G93A*^-expressing mice. To determine mouse genotype, RNA extraction and complimentary DNA (cDNA) synthesis was performed from tail biopsies acquired at postnatal day 28–40 followed by quantitative real-time PCR (qRT-PCR) using primers for the mutant G93A SOD1 gene (GGGAAGCTGTTGTCCCAAG and CAAGGGGAGGTAAAAGAGAGC). Both age- and litter-matched Wt and TgSOD1 male and female mice were used for all studies as described below.

### Behavior

All behavioral assessments, data collection, and analysis was carried out by an investigator who was blind to the experimental conditions (i.e., genotypes and treatment).

### Gait analysis

Manual gait analysis was performed using a limb painting procedure similar to previous studies^41,42^. Briefly, mice were first trained to traverse a horizontal corridor leading directly into their home cage by gentle nudges in the appropriate direction. On test days the bottoms of their hindlimbs were painted, by brushing with non-toxic food dye (Fisher Scientific), and the mice were allowed to walk the path to their home cage on a piece of paper. Three trials were performed at each experimental time point (P70, P90, P110, P130, P150). Stride length and width was determined by measuring the distance between the same points, on the ball mount region of the footprint, in two consecutive footprints and calculated from 2–3 hindpaw strides. Mean data from 4–6 strides across three trials was calculated.

### Rotarod

Mice were placed on a fixed speed (16 rpm) rotating rod (3.0 cm) (Rotamex, Columbus Instruments) as previously described^43,44^. Mice were trained to remain on the rotarod for 180 seconds once at P40. For each experimental time point (P70, P90, P110, P130, P150), the time mice spent on the rotating rod was calculated up to a maximum of 180 seconds. Three trials were performed for each time point and the greatest value for each session was used for analysis.

### Paw grip endurance test (PaGE)

The PaGE test was performed as previously described^43,45,46^. Briefly, mice were placed on the wire lid of a conventional housing cage that was inverted and held at ~45 cm above an open cage bottom. For experimental time points (P70, P90, P110, P130, P150), the time spent on the grid (before falling) was noted up to a maximum value of 90 seconds. The largest value from three individual trials was used for analysis.

### Weight and neurological scoring

Beginning at P50, weight and neurological score (using the ALS TDI criteria)^14,15^ were recorded for each mouse every 5 days until death or euthanasia. ALS TDI criteria are as follows:

Score of 0: Full extension of hind legs away from lateral midline when mouse is suspended by its tail, and mouse can hold this for two seconds, suspended two to three times.

Score of 1: Collapse or partial collapse of leg extension towards lateral midline (weakness) or trembling of hind legs during tail suspension.

Score of 2: Toes curl under at least twice during walking of 12 inches, or any part of foot is dragging along cage bottom/table.

Score of 3: Rigid paralysis or minimal joint movement, foot not being used for generating forward motion.

Score of 4: Mouse cannot right itself within 10 seconds after being placed on either side.

*Mice were euthanized upon obtaining a score of 4.

### Tissue dissection

Tissue was dissected from TgSOD1^*G93A*^ mice upon reaching a neurological score of 4^14,15^. Mice were sacrificed by administration of slow flow CO_2_ (10–30% of the chamber volume/minute) followed by immediate decapitation. Brain, gastrocnemius, and tibialis anterior tissue were removed and frozen in dry ice. Spinal cord was removed, frozen by gently lowering into the gas byproduct of liquid nitrogen and dissected into lumbar and non-lumbar regions. All tissue was stored at −80 °C prior to use. In addition, tail samples were extracted to perform a second round of confirmatory qRT-PCR for mouse genotyping. Tail samples were stored at −20 °C until used.

### Motor neuron quantification

Transverse sections (10 μm) of the lumbar spinal cord were cut from fresh frozen tissue. Hematoxylin and eosin (H&E) staining was performed on the tissue sections as previously described^15^. Within the region containing the ventral horn, all motor neurons are counted for three individual sections per animal (each separated by 50μm) to cover the areas of highest motor neuron density within the ventral horn. All counting was performed by an individual blinded to the genotypes. Images were acquired using a Zeiss microscope 20× objective (0.8NA) and processed with Metamorph image analysis software (Molecular Devices).

### Neuromuscular junction quantification

Longitudinal sections (20μm) of tibialis anterior muscle were made from fresh frozen tissue. Four to six sections for each specimen were stained with primary goat anti-VAChT (1:500; Millipore) and secondary Alexa Fluor 568 donkey anti-goat (1:1000; Invitrogen) antibodies along with an Alexa Fluor 488 conjugated to α-bungarotoxin (1:1000, Invitrogen). Sections were imaged with a 20× objective and analyzed by in-house software to determine the total number of VAChT and α-bungarotoxin counts per muscle along with the percentage VAChT/TMR-α-bungarotoxin colocalization per bilateral muscle sections per specimen as previously described^15^.

### Iba1 Immunohistochemical analysis

Frozen spinal cord sections were fixed in 4% PFA/PBS for 72 hours and dehydrated with 30% sucrose in PBS. Sections were washed three times (5 minutes each) with PBS and incubated with 3% H_2_O_2_ in PBS for 15 minutes to quench endogenous peroxidases. Sections were subsequently washed three times with PBS and blocked using 5% (v/v) normal goat serum (Vector Laboratories), 0.3% Triton X-100 in PBS. Primary antibody against Iba1 (rabbit polyclonal, 1:400, Wako, #019-19741) was diluted in a buffer containing 2.5% (v/v) normal goat serum, 0.3% Triton X-100 and incubated overnight at 4 °C. On the following day, samples were washed three times (10 minutes each) with PBS. The primary antibody was detected using a biotinylated secondary antibody (1:200) and VECTASTAIN Elite ABC HRP kit (Vector Laboratories), and developed with DAB (Vector Laboratories), following the provider’s instructions. Sections were dehydrated in a series of graded ethanol, cleared in xylene, and cover-slipped with Cytoseal-XYL xylene-based mounting medium (Thermo Fisher Scientific). Sections were imaged using a light microscope (TE360 Eclipse; Nikon, Japan) at 10× magnification. The Iba1^+^ cell area (%, area occupied by Iba1^+^ cells divided by the total area) was quantified for each spinal cord section using ImageJ software (Voxel counter plugin, NIH, USA). Two to three sections were analyzed per mouse. Values from each section were averaged to obtain a mean value for each animal.

### CD68 Immunohistochemical analysis

Frozen spinal cord sections were fixed in 4% PFA/PBS for 2 hours at room temperature. Sections were washed three times with PBS and incubated with 3% H_2_O_2_ in water for 20 minutes to quench endogenous peroxidases. Sections were subsequently washed three times with PBS and blocked using 5% (v/v) normal goat serum (Vector Laboratories), 0.2% Triton X-100 in PBS. Primary antibody against CD68 (rabbit polyclonal, 1:500, Abcam, ab125212) was diluted in a buffer containing 5% (v/v) normal goat serum, 0.2% Triton X-100 in PBS and incubated overnight at 4 °C. Next day, sections were washed three times with PBS/0.2% Triton X-100. The primary antibody was detected using a biotinylated secondary anti-rabbit antibody (1:300) and VECTASTAIN Elite ABC HRP kit (Vector Laboratories, PK-6101), and developed with DAB (Vector Laboratories), according to the manufacturer’s instructions. Sections were dehydrated in a series of graded ethanol, cleared in xylene, and cover-slipped with xylene-based mounting medium (Cytoseal-XYL, Thermo Fisher Scientific). Sections were imaged using a light microscope (TE360 Eclipse; Nikon, Japan) at 10x magnification. Two to three sections were analyzed per mouse and four to six images were acquired for each section. The CD68^+^ cell area (%, area occupied by CD68^+^ cells divided by the total area) was determined using ImageJ Software (Analyze Particles, NIH, USA). Values from each section were averaged to obtain a mean value for each animal.

### Immunofluorescence analysis of GFAP

Fresh frozen spinal cord tissue was sectioned at 10 μm in OCT Compound (Fisher). Section slides were equilibrated to room temperature then fixed in 4% paraformaldehyde in PBS for 20 minutes. Slides were then washed in 0.1% Triton-X in PBS for 20 minutes followed by an hour-long incubation with blocking buffer containing 1% BSA, 2% Normal Goat Serum, and 0.1% Triton-X in PBS. Sections were then incubated overnight in primary antibodie, GFAP (Cell Signaling 3670 S, 1:500), diluted in blocking buffer. After overnight incubation slides were rinsed in 0.1% Triton-X in PBS 3 × , followed by a two-hour incubation Cyanine (Cy3) conjugated secondary antibodies (Jackson ImmunoResearch, 1:1000). Slides were rinsed 3X in PBS and coverslipped with glass slides using ProLong Mounting Media with DAPI (Fisher Scientific). Four fields per animal were collected at 20X magnification on an Axio Imager microscope (ZEISS). For each field, intensity of staining above a constant threshold was measured. Average intensity from four fields was determined for each animal. These values were normalized to average intensity of Wt Vehicle above said threshold.

### Meso scale discovery multi-spot cytokine assay

Spinal cord frozen tissue was homogenized in ice-cold RIPA buffer (Thermo Fisher Scientific, #8990) supplemented with protease inhibitor cocktail (Thermo Fisher Scientific, #78430). Samples were centrifuged at 45,000 *g* for 30 minutes at 4 °C using an Optima TL ultracentrifuge and a TLA 120.2 rotor (Beckman Coulter). Expression levels of 10 pro-inflammatory cytokines and chemokines were assessed in the supernatants derived from spinal cord tissue or in the plasma, using an electrochemiluminescence-based multi-array method and MESO Quickplex SQ 120 system (MSD, Rockville, MD, USA). The 96-well V-PLEX Proinflammatory Mouse 1 Kit (Meso Scale Discovery, #K15048D) was used to measure simultaneously IFN-γ, IL-1β, IL-2, IL-4, IL-5, IL-6, CXCL1/KC/GRO, IL-10, IL-12, p70, and TNFα, following the manufacturer’s instructions. Briefly, samples were diluted in the calibrator and added to the plate coated with an array of cytokine capture antibodies. Samples were incubated in the plate for 2 hours with shaking at room temperature, followed by washes with the wash buffer provided in the kit. The detection antibody solution was added to each well and the plate was incubated for 2 hours. The plate was washed with the wash buffer and the 2× Read Buffer T was added. The signal was immediately measured on a MESO QuickPlex SQ 120 instrument and was analyzed using the DISCOVERY WORKBENCH 4.0 software (Meso Scale Diagnostics, LLC., Rockville, MD, USA). Protein concentrations in the supernatants or the plasma samples were measured using the Pierce BCA protein assay kit (Thermo Scientific). Values in the graphs represent levels of cytokines normalized to the corresponding protein concentrations.

### CCL2/MCP-1 ELISA assay

Spinal cord tissue was homogenized, and supernatants were derived as described in the section related to the Meso Scale Discovery cytokine assay. MCP-1 levels were measured in the supernatants generated from spinal cord tissue or in the plasma using the 96-well Mouse CCL2/JE/MCP-1 Quantikine ELISA Kit (R&D systems, #MJE00B), following the manufacturer’s instructions. Briefly, samples and diluted standards were added to the plate coated with MCP-1-specific antibody. Samples were incubated in the plate for 2 hours on a shaker at room temperature, followed by washes with the wash buffer provided in the kit. Subsequently, horseradish peroxidase conjugated antibody against MCP-1 was added to each well and incubated for 2 hours at room temperature on the shaker. The plate was washed with the wash buffer and incubated with Substrate solution for 30 minutes at room temperature. Next, Stop solution was added and the signal was read on a Microplate reader (Synergy 2, Biotek Instruments). The optical density was measured at 405 nm and was corrected with the optical density measured at 540 nm. MCP-1 levels in the samples were calculated based on the MCP-1 standard curve. Protein concentrations in the supernatants were measured by the Pierce BCA protein assay kit (Thermo Scientific). Values in the graphs represent levels of MCP-1 normalized to the corresponding protein concentrations.

### Mast cell analysis

Mast cell counts and degranulation was assessed based on previously published methods^37^. Briefly, longitudinal sections (20um) of tibialis anterior muscle were made from fresh frozen tissue. Sections were washed and hydrated 2 times in distilled water for 10 minutes and embedded in 1% toluidine blue solution (Fisher Scientific, T16125) for 10–20 minutes. Slides were washed in distilled water and dehydrated in 70% ethanol, 95% ethanol, and in 100% ethanol. Slides were cleared in xylene and were mounted in ProLong mounting media (Invitrogen). Images were acquired using a Zeiss Axioscope light microscope. The number of toluidine blue-positive metachromatic mast cells were counted in six individual entire muscle section for each animal. The number of degranulating mast cells was also counted in each of these sections for each animal and were identified by extensive metachromatic granules being released by an isolated cell as described previously^37^.

### Statistics

Data in the main text are presented as median values. Box plots are used for graphical representation of population data with the central line representing the median, the edges representing the interquartile ranges, and the whiskers representing 10–90th percentiles. Data are also represented as medians + interquartile ranges or percent values. Sample sizes are included in the figure legends. Comparisons for unrelated samples were performed using a two-way ANOVA followed by Tukey’s or Sidak’s multiple comparison’s test or a one-way ANOVA test followed by Tukey’s multiple comparison post-tests at a significance level (α) of 0.05. For p < 0.05 and >0.00001, exact P values (two-tailed) are reported. Kruskal-Wallis ANOVA followed by Dunn’s post-hoc test was used for data that was not normally distributed.

### Ethical approval

All animal care, husbandry and experimentation were performed according to the guidelines set by the Massachusetts General Hospital Subcommittee on Research Animal Care. All animal studies were approved by the Massachusetts General Hospital Institutional Animal Care and Use Committee (2014N000018).

## Supplementary information

Supplementary Data

## Data Availability

The datasets generated during and/or analyzed during the current study are available from the corresponding author on reasonable request.]
