# Supplementary material for: Cromolyn sodium delays disease onset and is neuroprotective in the SOD1G93A Mouse Model of amyotrophic lateral sclerosis
Source: Sci Rep. 2019 Nov 27;9:17728. doi: 10.1038/s41598-019-53982-w (PMC6881366; doi:10.1038/s41598-019-53982-w)
Supplement: Supplementary file 1 — Supplementary Data [file 41598_2019_53982_MOESM1_ESM.pdf]

Cromolyn sodium delays disease onset and is neuroprotective in the SOD1^{G93A} Mouse Model of amyotrophic lateral sclerosis

Eric J. Granucci*[1], Ana Griciuc*[2], Kaly A. Mueller*[1], Alexandra N. Mills[1], Hoang Le[2], Amanda M. Dios[1], Danielle McGinty[2], Joao Pereira[1], David Elmaleh[3], James D. Berry[1], Sabrina Paganoni[1,4], Merit E. Cudkowicz[1], Rudolph E. Tanzi[2], Ghazaleh Sadri-Vakili[1]

* These authors contributed equally

[1] Healey Center for ALS at MassGeneral Hospital, Massachusetts General Hospital, Boston, MA, USA

[2] Genetics and Aging Research Unit, McCance Center for Brain Health, MassGeneral Institute for Neurodegenerative disease, Department of Neurology, Massachusetts General Hospital and Harvard Medical School, Charlestown, MA 02129, USA

[3] AZTherapies, Boston, MA

[4] Spaulding Rehabilitation Hospital, Department of PM&R, Harvard Medical School

Corresponding author:
Ghazaleh Sadri-Vakili, Ph.D., MassGeneral Institute for Neurodegenerative Disease, Massachusetts General Hospital, Bldg 114 16th Street, R2200, Charlestown, MA 02129
Telephone: 617-724-1487; Fax: 617-724-1480; Email: gsadrivakili@mgh.harvard.edu

Keywords: ALS, inflammation, microglia, cromolyn, treatment

Running Title: Cromolyn is neuroprotective in SOD1^{G93A} mice

Supplementary Data

The effect of cromolyn sodium treatment on female and male body weight

We also assessed the effect of treatment on body weight in female and male mice separately. Two-way ANOVA demonstrated a significant effect of age [F(9, 524) = 5.686, p < 0.0001], treatment [F(3, 524) = 13.76, p < 0.0001], and age X treatment interaction effect [F(27, 524) = 4.578, p < 0.0001] on body weight in female mice. Tukey's multiple comparison test revealed that there was a significant decrease in body weight in the TgSOD1-Vehicle group compared to wild-type groups at P120 and P130 (S2b). There was also a significant decrease in body weight in the TgSOD1-Cromolyn group compared to wild-type groups at P130, P140, and P150. Two-way ANOVA in male mice revealed a significant effect of age [F(8, 549) = 7.11, p < 0.0001], treatment [F(3, 549) = 58.48, p < 0.0001], and age X treatment interaction effect [F(24, 549) = 3.623, p < 0.0001] on body weight in male mice. Tukey's multiple comparison test revealed that there was a significant decrease in body weight in the male TgSOD1-Vehicle group compared to wild-type groups at P90, P100, P110, P120, P130, and P140. Thus, cromolyn treatment did not impact body weight of TgSOD1 mice, except for at P130 when a significant improvement was observed.

The effect of cromolyn sodium treatment on female and male PAGE task, rotarod, and gait performance

Two-way ANOVA demonstrated a significant effect of age [F(4, 274) = 41.14, p < 0.0001], treatment [F(3, 274) = 53.41, p < 0.0001] and a significant age X treatment interaction on PaGE performance in female mice [F(12, 274) = 16.2, p < 0.0001] (Figure S3a). Tukey's post-hoc analysis revealed a significant decrease in PaGE performance in the TgSOD1-Vehicle group at P120 compared to Wt-Vehicle and Wt-Cromolyn groups (Figure S3a). In addition, there was a significant decrease in PaGE performance in the TgSOD1-Cromolyn group compared to both wild-type groups at P100 and P120. Importantly, there was a statistically significant difference between TgSOD1-Vehicle and TgSOD1-Cromolyn at P100 where the cromolyn treated group demonstrated greater deficits in PaGE performance (Figure S3a). In male mice, two-way ANOVA demonstrated a significant effect of age [F(3, 208) = 13.34, p < 0.0001], treatment [F(3, 208) = 48, p < 0.0001] and a significant age X treatment interaction on PaGE [F(9, 208) = 5.828, p < 0.0001] (Figure S3b). Tukey's post-hoc analysis revealed a significant decrease in PaGE in the TgSOD1-Vehicle group compared to both wild-type groups at P80, P100, and P120. There was also a significant decrease in PaGE in the TgSOD1-Cromolyn group compared to both wild-type groups at P100 and P120. Importantly, there was a significant improvement in PaGE at P120 between the two male transgenic groups (Figure S3b). Thus, cromolyn treatment improved PaGE performance in treated TgSOD1 mice as compared to the vehicle treated TgSOD1 group.

In female mice, two-way ANOVA demonstrated a significant effect of age [F(2, 176) = 19.04, p < 0.0001] and treatment [F(3, 176) = 14.48, p < 0.0001] in rotarod performance. However, there was no significant age X treatment interaction on rotarod performance [F(6, 176) = 0.498, p=0.8086]. Tukey's post-hoc analysis revealed a significant difference between TgSOD1-Vehicle with both Wt-Vehicle and Wt-Cromolyn at P70, P90 and P120 (Figure S4b). Similarly, post-hoc analysis revealed a significant decrease in rotarod performance between

TgSOD1-Cromolyn group compared to Wt-Vehicle and Wt-Cromolyn at all time points (Figure S4b). In male mice, two-way ANOVA demonstrated a significant effect of age [$F(2, 169) = 9.97$, $p < 0.0001$] and treatment [$F(3, 169) = 28.15$, $p < 0.0001$]. However, there was no significant age X treatment interaction on rotarod performance [$F(6, 169) = 0.561$, $p=0.7604$]. Tukey's post-hoc analysis revealed a significant difference between TgSOD1-Vehicle with both Wt-Vehicle and Wt-Cromolyn at P70, P90 and P120 in male treated mice (Figure S4c). Similarly, post-hoc analysis revealed a significant decrease in rotarod performance between male TgSOD1-Cromolyn group compared to Wt-Vehicle and Wt-Cromolyn at all time points (Figure S4c). These data indicate that cromolyn treatment did not alter rotarod performance in TgSOD1 mice.

In female mice, two-way ANOVA revealed a significant effect of age [$F(2, 190) = 27.85$, $p < 0.0001$], treatment [$F(3, 190) = 8.389$, $p < 0.0001$], and age X treatment interaction on stride length [$F(6, 190) = 6.278$, $p < 0.0001$]. Tukey's post-hoc analysis revealed a significant decrease in stride length in TgSOD1-Vehicle and TgSOD1-Cromolyn treated female mice compared with both wild-type groups at P120 (Figure S5b). Similarly, in male mice, two-way ANOVA revealed a significant effect of age [$F(2, 205) = 37.9$, $p < 0.0001$], treatment [$F(3, 205) = 10.84$, $p < 0.0001$], and age X treatment interaction on stride length [$F(6, 205) = 10.86$, $p < 0.0001$]. Tukey's post-hoc analysis revealed a significant decrease in stride length in male TgSOD1-Vehicle and TgSOD1-Cromolyn treated mice compared with both wild-type groups at P120 (Figure S5c). In addition, alterations in stride width were also assessed following treatment. In all groups, two-way ANOVA revealed that while there was no effect of treatment, there was a significant effect of age [$F(2, 397) = 18.3$, $p < 0.0001$] and age X treatment [$F(6, 397) = 3.159$, $p = 0.0049$] on stride width. Tukey's post-hoc analysis revealed a significant increase in stride width at P120 in TgSOD1-Vehicle group compared to Wt-Vehicle (Figure S6a). Two-way ANOVA analysis of female mice alone revealed a significant effect on age [$F(2, 1935) = 5.837$, $p = 0.0035$] only (Figure S6b). Furthermore, two-way ANOVA in male mice revealed that while there was no effect of treatment, there was a significant effect of age [$F(2, 189) = 14.84$, $p < 0.0001$] and age X treatment [$F(6, 189) = 3.978$, $p = 0.0009$] on stride width. Post-hoc analysis revealed a significant increase in stride width in the TgSOD1-Vehicle treated mice compared to both wild-type groups (Figure S6c). Thus, cromolyn treatment did not alter gait performance (e.g. stride length or width) in TgSOD1 mice.

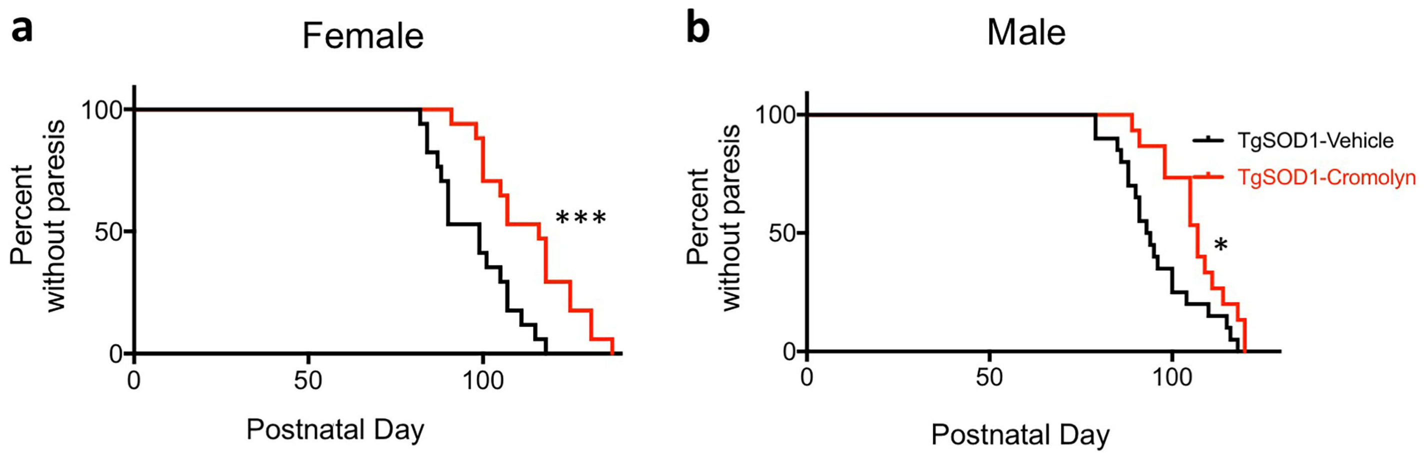

Suppl. Fig 1. ***Cromolyn sodium treatment delayed the age at paresis onset in TgSOD1 mice.***

a) In female mice there was a significant delay in the onset of motor symptoms following cromolyn treatment (Mantel-Cox test) c) In male mice cromolyn treatment significantly delayed the onset of motor symptoms (Mantel-Cox test). Female mice: TgSOD1-Vehicle (n = 19; black), and TgSOD1-Cromolyn (n = 17; red). Male mice: TgSOD1-Vehicle (n = 21; black), TgSOD1-Cromolyn (n = 17; red). * denotes differences between TgSOD1-Vehicle and Tg-SOD1-Cromolyn. * $p < 0.05$; *** $p < 0.001$.

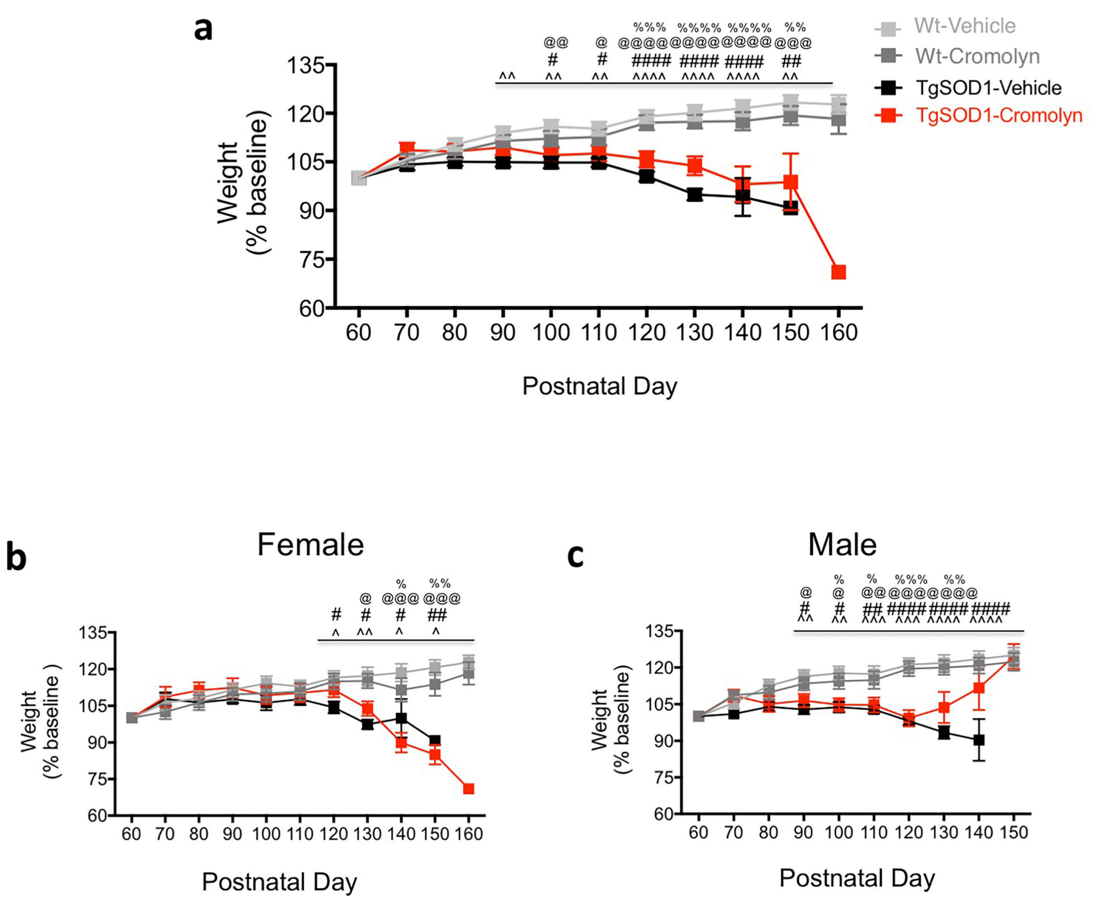

Suppl. Fig 2. ***Cromolyn sodium treatment does not alter body weight of TgSOD1 mice.*** a) Two-way ANOVA and Tukey's multiple comparison test revealed a significant improvement in body weight in the TgSOD1-Cromolyn group compared to TgSOD1-Vehicle group at P130 only. There was also a significant decrease in body weight in the TgSOD1-Vehicle group compared to Wt-Vehicle and Wt-Cromolyn at P100, P110, P120, P130, P140, and P150. There was also a significant decrease in body weight in the TgSOD1-Cromolyn group compared to Wt-Cromolyn group at P100, P110, P120, P130, P140, and P150. There was a significant difference in body weight between TgSOD1-Cromolyn group and Wt-Vehicle group at P120, P130, and P140 only. b) In female mice, two-way ANOVA and Tukey's multiple comparison test revealed a significant decrease in body weight in the TgSOD1-Vehicle group compared to wild-type groups at P120 and P130. There was also a significant decrease in body weight in the TgSOD1-Cromolyn group compared to wild-type groups at P130, P140, and P150. c) In male mice, two-way ANOVA and Tukey's multiple comparison test revealed a significant decrease in body weight in the TgSOD1-Vehicle group compared to wild-type groups at P90, P100, P110, P120, P130, and P140. There was also a significant decrease in body weight in the TgSOD1-Cromolyn group compared to wild-type groups at P90, P100, P110, P120, and P130. Female mice: Wt-Vehicle (n = 19; light grey), Wt-Cromolyn (n = 17; dark grey), TgSOD1-Vehicle (n = 19; black), and TgSOD1-Cromolyn (n = 17; red). Male mice: Wt-Vehicle (n = 18; light grey), Wt-Cromolyn (n = 21; dark grey), TgSOD1-Vehicle (n = 21; black), TgSOD1-Cromolyn (n = 17; red). * denotes differences between TgSOD1-Vehicle and Tg-SOD1-Cromolyn; ^ denotes differences between TgSOD1-Vehicle and Wt-Vehicle; # denotes differences between TgSOD1-Vehicle and Wt-

Cromolyn; @ denotes differences between TgSOD1-Cromolyn and Wt-Vehicle; % denotes differences between TgSOD1-Cromolyn and Wt-Cromolyn. * $p < 0.05$; ** $p < 0.01$; *** $p < 0.001$; **** $p < 0.0001$, the same statistical significance is associated with each symbol. Data are presented as median and interquartile ranges.

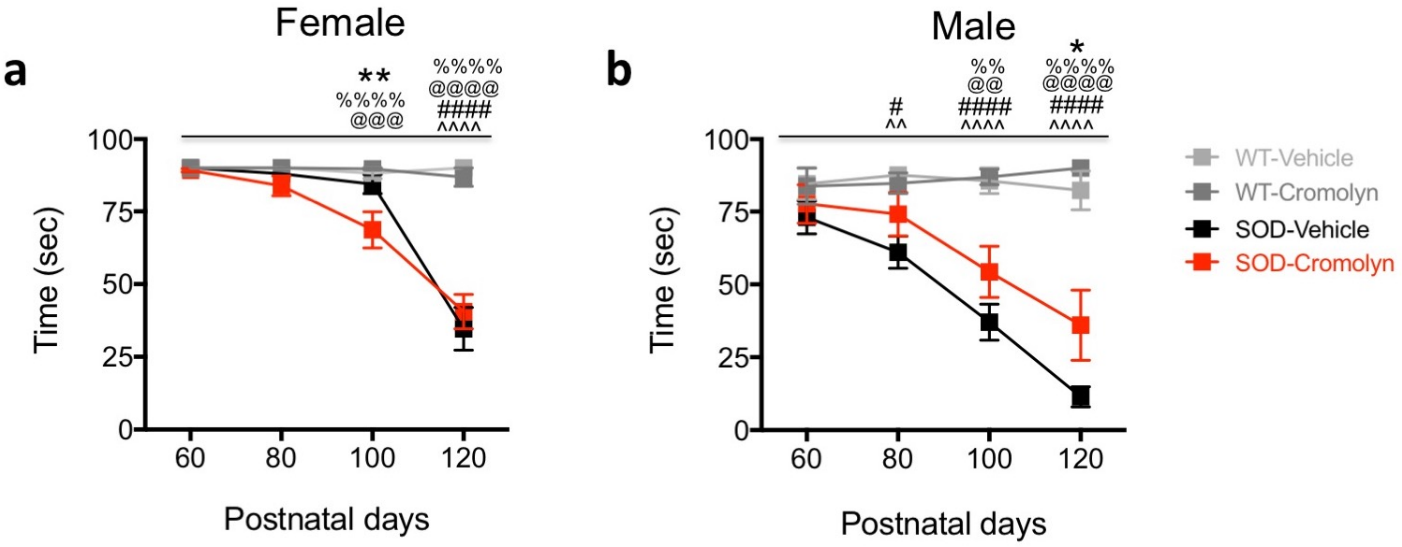

Suppl. Fig 3. ***Cromolyn sodium treatment improved performance on the PAGE task in TgSOD1 mice*** a) In female mice, two-way ANOVA and Tukey's post-hoc analysis revealed a significant decrease in PaGE in the TgSOD1-Vehicle group at P120 compared to Wt-Vehicle and Wt-Cromolyn groups. In addition, there was a significant decrease in PaGE in the TgSOD1-Cromolyn group compared to both wild-type groups at P100 and P120. Importantly, there was a significant difference between the two transgenic groups at P100 with a worsening in the cromolyn treated female group. b) In male mice, two-way ANOVA and Tukey's post-hoc analysis revealed a significant decrease in PaGE in the TgSOD1-Vehicle group compared to both wild-type groups at P80, P100, and P120. There was also a significant decrease in PaGE in the TgSOD1-Cromolyn group compared to both wild-type groups at P100 and P120. Importantly, there was a significant improvement in PaGE at P120 between the two male transgenic groups. Female mice: Wt-Vehicle (n = 19; light grey), Wt-Cromolyn (n = 17; dark grey), TgSOD1-Vehicle (n = 19; black), and TgSOD1-Cromolyn (n = 17; red). Male mice: Wt-Vehicle (n = 18; light grey), Wt-Cromolyn (n = 21; dark grey), TgSOD1-Vehicle (n = 21; black), TgSOD1-Cromolyn (n = 17; red). Data are presented as median and interquartile ranges. * denotes differences between TgSOD1-Vehicle and Tg-SOD1-Cromolyn; ^ denotes differences between TgSOD1-Vehicle and Wt-Vehicle; # denotes differences between TgSOD1-Vehicle and Wt-Cromolyn; @ denotes differences between TgSOD1-Cromolyn and Wt-Vehicle; % denotes differences between TgSOD1-Cromolyn and Wt-Cromolyn. * $p < 0.05$; ** $p < 0.01$; *** $p < 0.001$; **** $p < 0.0001$, the same statistical significance is associated with each symbol. Data are presented as median and interquartile ranges.

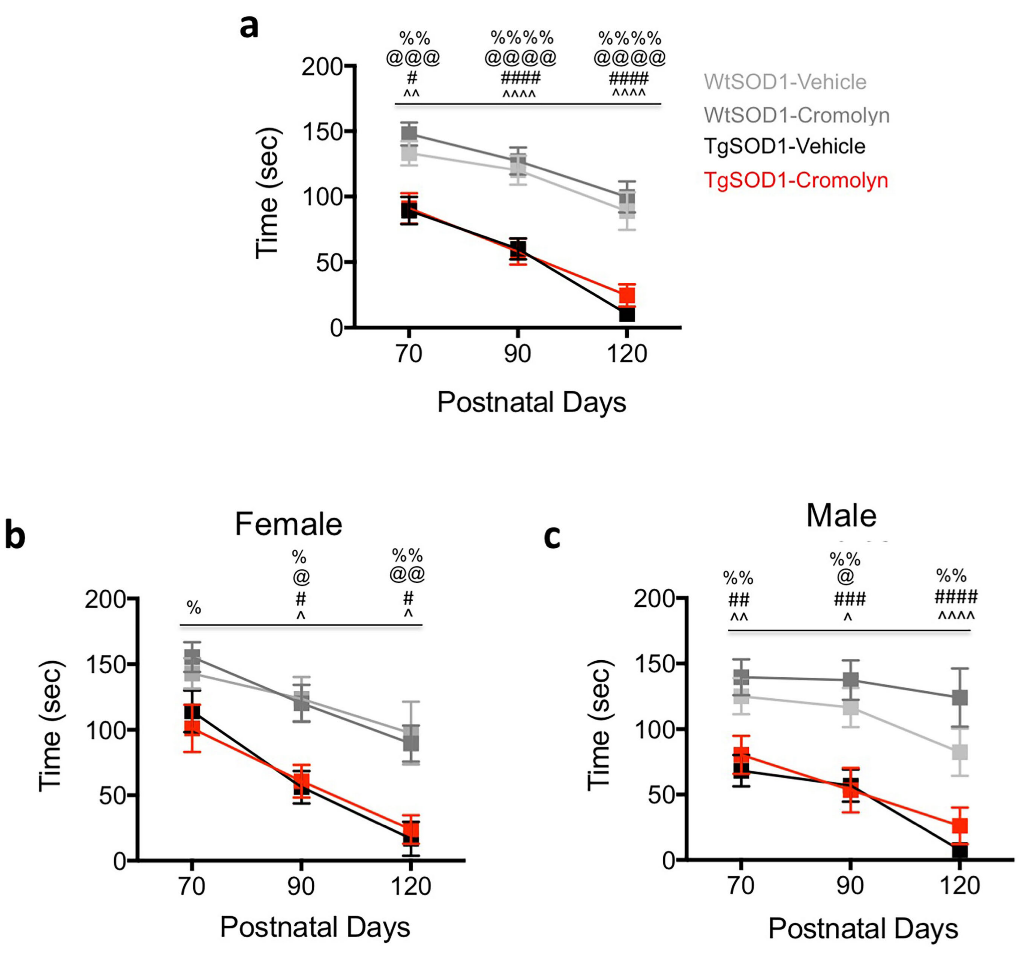

Suppl. Fig 4. ***Cromolyn sodium did not alter performance on the rotarod*** a) Two-way ANOVA and Tukey's post-hoc analysis revealed no difference in rotarod performance between the TgSOD1-Vehicle and TgSOD1-Cromolyn mice. There was a significant difference between TgSOD1-Vehicle with both Wt-Vehicle and Wt-Cromolyn at P70, P90 and P120. Similarly, post-hoc analysis revealed a significant decrease in rotarod performance between TgSOD1-Cromolyn group compared to Wt-Vehicle and Wt-Cromolyn at all time points. b) In female mice, two-way ANOVA and Tukey's post-hoc analysis revealed a significant difference between TgSOD1-Vehicle and both Wt-Vehicle and Wt-Cromolyn at P70, P90 and P120. Similarly, post-hoc analysis revealed a significant decrease in rotarod performance between TgSOD1-Cromolyn group compared to Wt-Vehicle and Wt-Cromolyn at all time points c) In male mice, two-way ANOVA and Tukey's post-hoc analysis revealed a significant difference between TgSOD1-Vehicle with both Wt-Vehicle and Wt-Cromolyn at P70, P90 and P120 in male treated mice. Similarly, post-hoc analysis revealed a significant decrease in rotarod performance between male TgSOD1-Cromolyn group compared to Wt-Vehicle and Wt-Cromolyn at all time points (Figure 4c). Female mice: Wt-Vehicle (n = 19; light grey), Wt-Cromolyn (n = 17; dark grey), TgSOD1-Vehicle (n = 19; black), and TgSOD1-Cromolyn (n = 17; red). Male mice: Wt-Vehicle (n = 18; light grey), Wt-Cromolyn (n = 21; dark grey), TgSOD1-Vehicle (n = 21; black), TgSOD1-Cromolyn (n = 17; red). * denotes differences between TgSOD1-Vehicle and Tg-SOD1-Cromolyn; ^ denotes differences between TgSOD1-Vehicle and Wt-Vehicle; # denotes differences between TgSOD1-Vehicle and Wt-Cromolyn; @ denotes differences

between TgSOD1-Cromolyn and Wt-Vehicle; % denotes differences between TgSOD1-Cromolyn and Wt-Cromolyn. * $p < 0.05$; ** $p < 0.01$; *** $p < 0.001$; **** $p < 0.0001$, the same statistical significance is associated with each symbol. Data are presented as median and interquartile ranges.

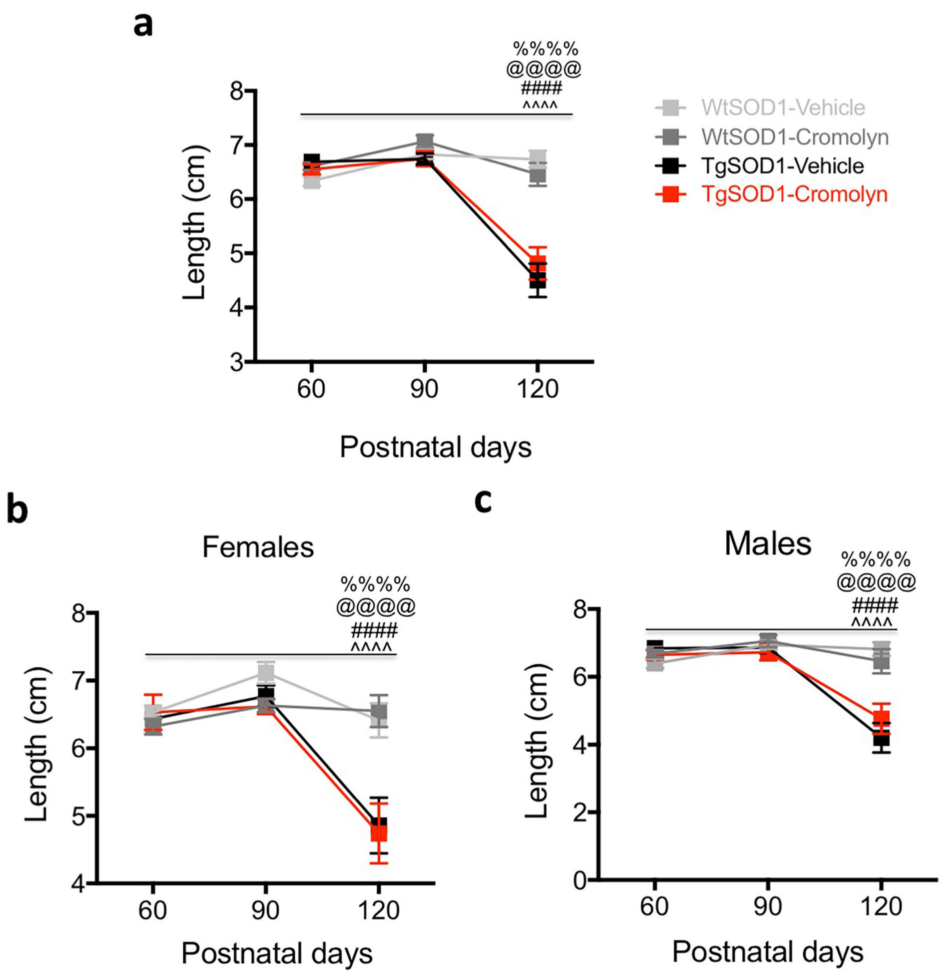

Suppl. Fig 5. ***Cromolyn sodium did not alter stride length in TgSOD1 mice.***

a) Two-way ANOVA and Tukey's post-hoc analysis revealed no significant difference in stride length between TgSOD1-cromolyn and TgSOD1-Vehicle groups. There was a significant decrease in stride length in TgSOD1-Vehicle compared with both wild-type groups at P120. Similarly, post-hoc analysis revealed a significant decrease in stride length in TgSOD1-Cromolyn group compared wild-type mice at P120. b) In female mice, two-way ANOVA and Tukey's post-hoc analysis revealed a significant decrease in stride length in TgSOD1-Vehicle and TgSOD1-Cromolyn treated female mice compared with both wild-type groups at P120. c) Similarly, in male mice, two-way ANOVA and Tukey's post-hoc analysis revealed a significant decrease in stride length in male TgSOD1-Vehicle and TgSOD1-Cromolyn treated mice compared with both wild-type groups at P120. Female mice: Wt-Vehicle (n = 19; light grey), Wt-Cromolyn (n = 17; dark grey), TgSOD1-Vehicle (n = 19; black), and TgSOD1-Cromolyn (n = 17; red). Male mice: Wt-Vehicle (n = 18; light grey), Wt-Cromolyn (n = 21; dark grey), TgSOD1-Vehicle (n = 21; black), TgSOD1-Cromolyn (n = 17; red). * denotes differences between TgSOD1-Vehicle and Tg-SOD1-Cromolyn; ^ denotes differences between TgSOD1-Vehicle and Wt-Vehicle; # denotes differences between TgSOD1-Vehicle and Wt-Cromolyn; @ denotes differences between TgSOD1-Cromolyn and Wt-Vehicle; % denotes differences between TgSOD1-Cromolyn and Wt-Cromolyn. *** p < 0.001; **** p <

0.0001, the same statistical significance is associated with each symbol. Data are presented as median and interquartile ranges.

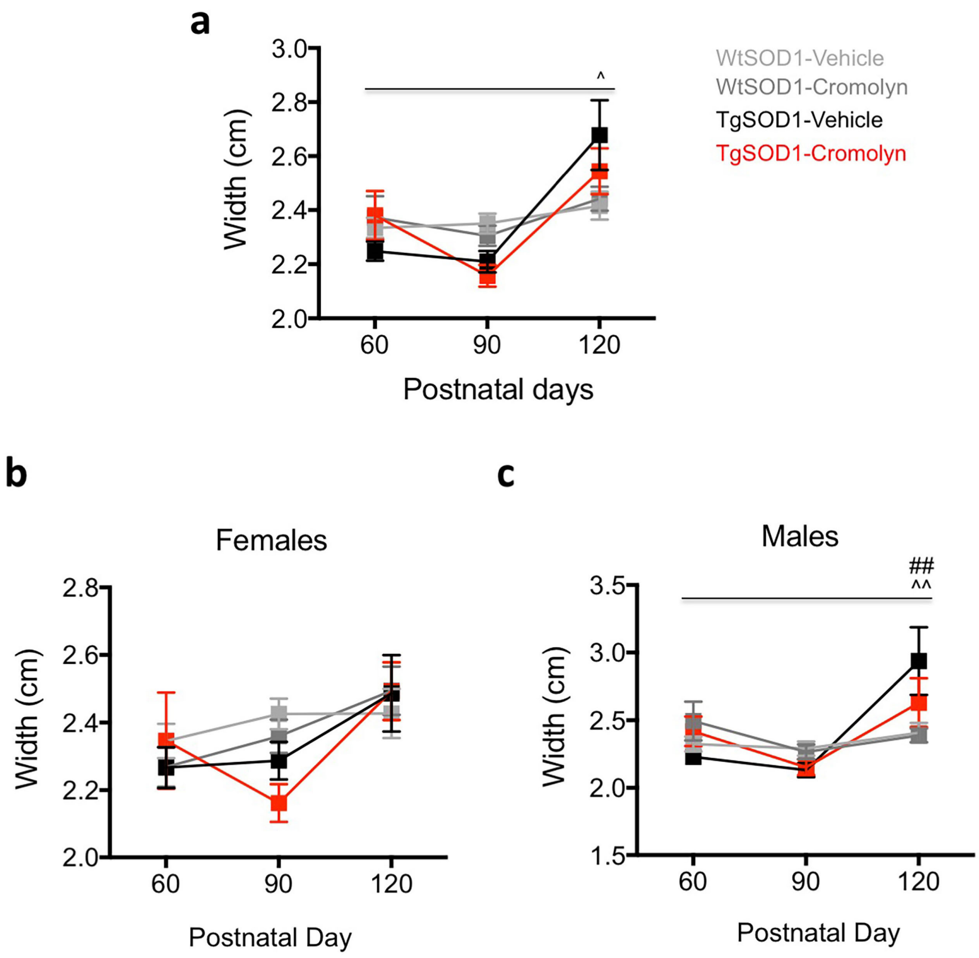

Suppl. Fig 6. ***Cromolyn sodium did not alter stride width in TgSOD1 mice.***

a) Two-way ANOVA revealed that while there was no effect of treatment, there was a significant effect of age and age X treatment on stride width. Post-hoc analysis revealed a significant increase in stride width in the TgSOD1-Vehicle treated mice compared to both wild-type groups Tukey's post-hoc analysis revealed a significant increase in stride width at P120 in TgSOD1-Vehicle group compared to Wt-Vehicle. b) Two-way ANOVA analysis of female mice alone revealed a significant effect on age only. c) Two-way ANOVA in male mice revealed that while there was no effect of treatment, there was a significant effect of and age X treatment on stride width. Female mice: Wt-Vehicle (n = 19; light grey), Wt-Cromolyn (n = 17; dark grey), TgSOD1-Vehicle (n = 19; black), and TgSOD1-Cromolyn (n = 17; red). Male mice: Wt-Vehicle (n = 18; light grey), Wt-Cromolyn (n = 21; dark grey), TgSOD1-Vehicle (n = 21; black), TgSOD1-Cromolyn (n = 17; red). * denotes differences between TgSOD1-Vehicle and Tg-SOD1-Cromolyn; ^ denotes differences between TgSOD1-Vehicle and Wt-Vehicle; # denotes differences between TgSOD1-Vehicle and Wt-Cromolyn; @ denotes differences between TgSOD1-Cromolyn and Wt-Vehicle; % denotes differences between TgSOD1-Cromolyn and Wt-Cromolyn. * $p < 0.05$; ** $p < 0.01$, the same statistical significance is associated with each symbol. Data are presented as median and interquartile ranges.

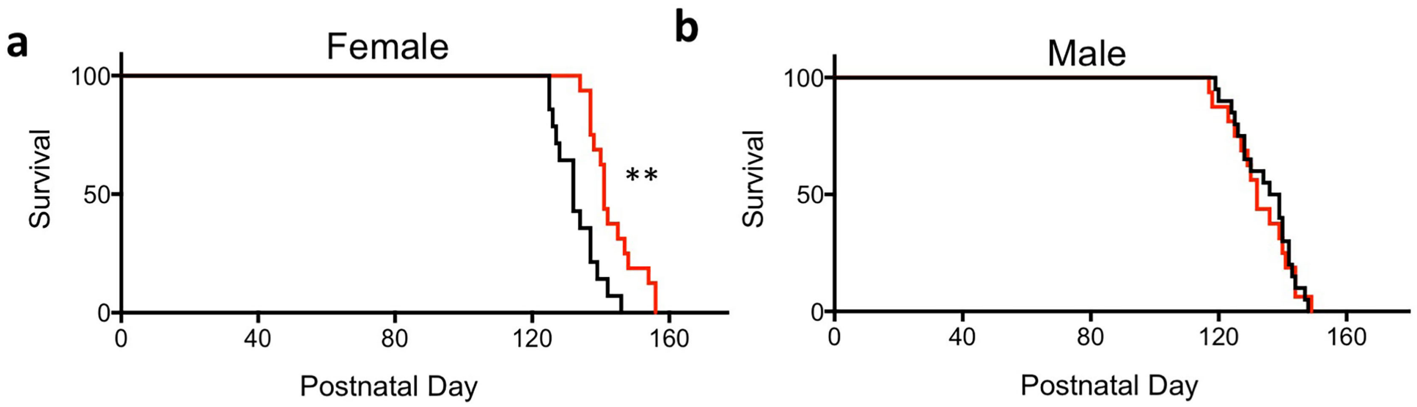

Suppl. Fig 7. ***Cromolyn sodium increased survival in female TgSOD1 mice.***

a) There was a significant effect of treatment on survival in female mice only (Mantel-Cox test) b) There was no effect of treatment on male mice. Female mice: TgSOD1-Vehicle (n = 19; black), and TgSOD1-Cromolyn (n = 17; red). Male mice: TgSOD1-Vehicle (n = 21; black), TgSOD1-Cromolyn (n = 17; red). * denotes differences between TgSOD1-Vehicle and Tg-SOD1-Cromolyn. ** $p < 0.01$.

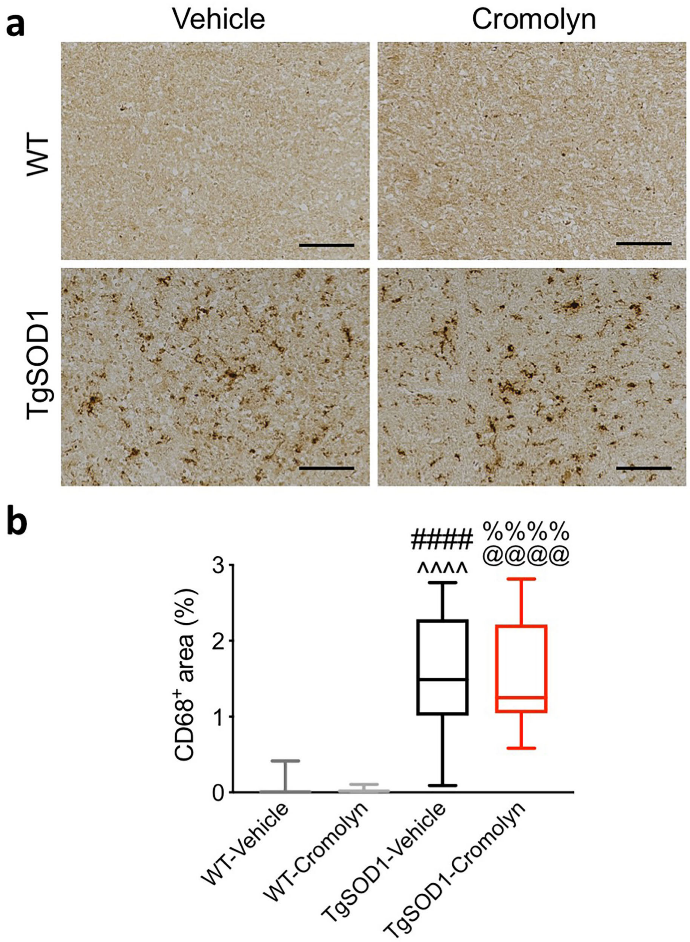

Suppl. Fig S8. ***Cromolyn treatment does not alter CD68⁺ microglial cell area in the spinal cord of TgSOD1 mice.*** a) CD68⁺ microglia of the lumbar spinal cord were visualized using the CD68-specific antibody and DAB staining. Images of spinal cord fields from mice of indicated genotypes and treatment. b) Quantifications of CD68⁺ cell area revealed no difference in expression of CD68 in TgSOD1-Cromolyn mice compared to TgSOD1-Vehicle. There was a significant increase in CD68⁺ cell area (%) in the spinal cord of both TgSOD1-Vehicle and TgSOD1-Cromolyn compared to both wild-type groups as demonstrated two-way ANOVA and Tukey's post-hoc analysis. Wt-Vehicle (n = 11; light grey), Wt-Cromolyn (n = 12; dark grey), TgSOD1-Vehicle (n = 19; black), and TgSOD1-Cromolyn (n = 14; red). * denotes differences between TgSOD1-Vehicle and Tg-SOD1-Cromolyn; ^ denotes differences between TgSOD1-Vehicle and Wt-Vehicle; # denotes differences between TgSOD1-Vehicle and Wt-Cromolyn; @ denotes differences between TgSOD1-Cromolyn and Wt-Vehicle; % denotes differences between TgSOD1-Cromolyn and Wt-Cromolyn. **** $p < 0.0001$, the same statistical significance is associated with each symbol. Data are presented as median and interquartile ranges. Scale bar = 100μm.

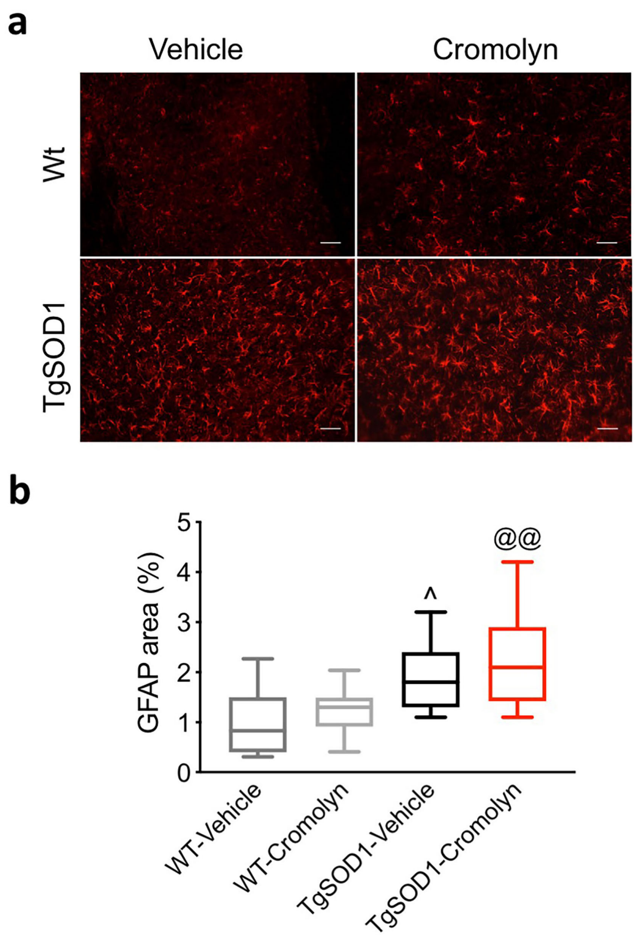

Suppl. Fig S9. ***Cromolyn treatment does not alter astrogliosis in the spinal cord of TgSOD1 mice.*** a) Astrocytes in the lumbar spinal cord were visualized using the GFAP antibody. Images of spinal cord fields from mice of indicated genotypes and treatment. b) Quantifications of GFAP cell area revealed no difference in expression of GFAP in TgSOD1-Cromolyn mice compared to TgSOD1-Vehicle. There was a significant increase in GFAP cell area (%) in the spinal cord of both TgSOD1-Vehicle and TgSOD1-Cromolyn compared to both wild-type groups as demonstrated two-way ANOVA and Tukey's post-hoc analysis. Wt-Vehicle (n = 11; light grey), Wt-Cromolyn (n = 12; dark grey), TgSOD1-Vehicle (n = 19; black), and TgSOD1-Cromolyn (n = 14; red). * denotes differences between TgSOD1-Vehicle and Tg-SOD1-Cromolyn; ^ denotes differences between TgSOD1-Vehicle and Wt-Vehicle; # denotes differences between TgSOD1-Vehicle and Wt-Cromolyn; @ denotes differences between TgSOD1-Cromolyn and Wt-Vehicle; % denotes differences between TgSOD1-Cromolyn and Wt-Cromolyn. * $p < 0.05$; ** $p < 0.01$, the same statistical significance is associated with each symbol. Data are presented as median and interquartile ranges. Scale bar = 50μm.

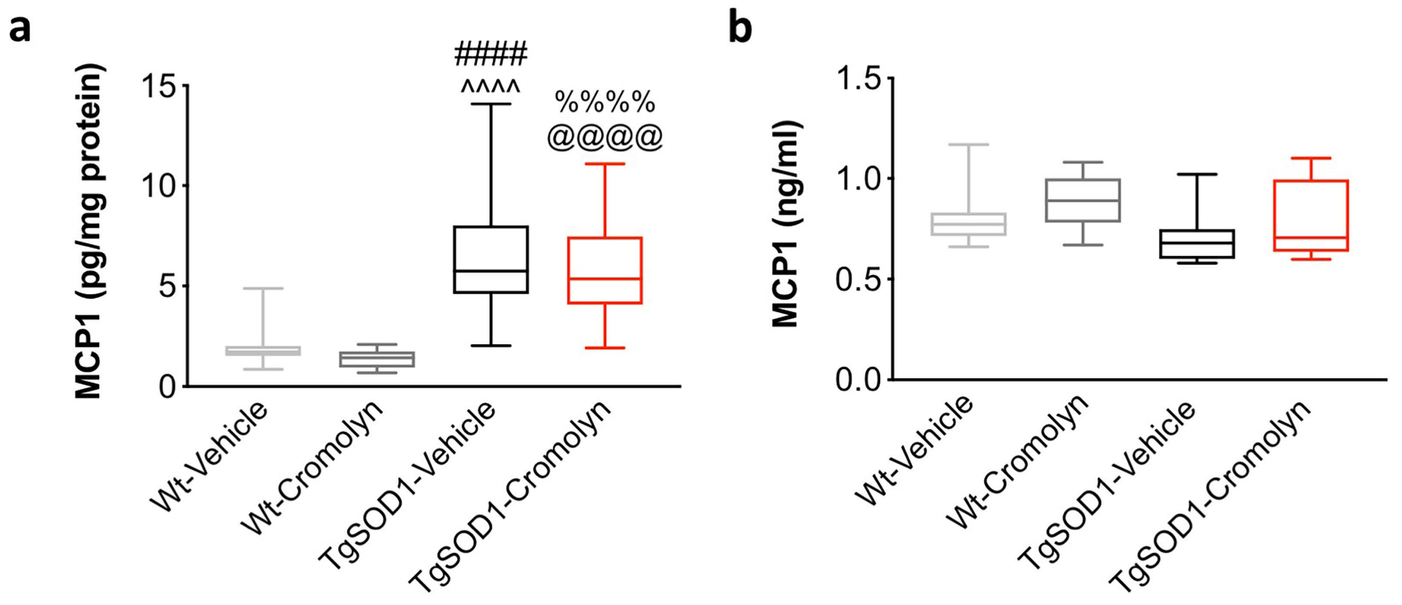

Suppl. Fig S10. ***Cromolyn treatment does not alter MCP-1 levels in the spinal cord or plasma of TgSOD1 mice.*** a) Two-way ANOVA and Tukey's post-hoc analysis revealed a significant increase in MCP-1 levels in the spinal cord of TgSOD1-Vehicle mice compared to both Wt-Vehicle and Wt-Cromolyn groups. However, there was no effect of cromolyn treatment on MCP-1 levels in the spinal cord of TgSOD1-Cromolyn compared to TgSOD1-Vehicle. b) One-way ANOVA and Tukey's post-hoc analysis revealed that MCP-1 levels were not altered in the plasma in any of the groups. For spinal cord, Wt-Vehicle (n = 15; light grey), Wt-Cromolyn (n = 19; dark grey), TgSOD1-Vehicle (n = 17; black), and TgSOD1-Cromolyn (n = 17; red). For plasma, Wt-Vehicle (n = 11; light grey), Wt-Cromolyn (n = 11; dark grey), TgSOD1-Vehicle (n = 9; black), and TgSOD1-Cromolyn (n = 9; red). * denotes differences between TgSOD1-Vehicle and Tg-SOD1-Cromolyn; ^ denotes differences between TgSOD1-Vehicle and Wt-Vehicle; # denotes differences between TgSOD1-Vehicle and Wt-Cromolyn; @ denotes differences between TgSOD1-Cromolyn and Wt-Vehicle; % denotes differences between TgSOD1-Cromolyn and Wt-Cromolyn. **** $p < 0.0001$, the same statistical significance is associated with each symbol. Data are presented as median and interquartile ranges.
